# Dengue Virus Inhibitors as Potential Broad-Spectrum Flavivirus Inhibitors

**DOI:** 10.3390/ph18030283

**Published:** 2025-02-20

**Authors:** Larisa Ivanova, Krystyna Naumenko, Margus Varjak, Sandra Koit, Yehudit Morozovsky, Andres Merits, Mati Karelson, Eva Zusinaite

**Affiliations:** 1 Institute of Chemistry, University of Tartu, Ravila 14A, 50411 Tartu, Estonia; larisa.ivanova@ut.ee (L.I.); yehudit.morozovsky@ut.ee (Y.M.); 2 Institute of Bioengineering, University of Tartu, Nooruse 1, 50411 Tartu, Estonia; krystyna.naumenko@ut.ee (K.N.); sandra77@ut.ee (S.K.); andres.merits@ut.ee (A.M.); 3 Zabolotny Institute of Microbiology and Virology of NASU, 154 Acad. Zabolotny St., Kyiv 03143, Ukraine; 4 Institute of Technology, University of Tartu, Nooruse 1, 50411 Tartu, Estonia; margus.varjak@ut.ee

**Keywords:** flaviviruses, broad-spectrum inhibitors, molecular dynamics simulation

## Abstract

**Background.** Flaviviruses spread from endemic to non-endemic areas, causing illness in millions of people worldwide. The lack of effective therapies and the rapid expansion of flaviviral infections worldwide emphasize the importance of finding effective antivirals to treat such diseases. **Objectives.** To find out the potential broad-spectrum flavivirus inhibitors among previously reported inhibitors of DENV2/DENV4. **Methods.** The cytotoxicity of compounds was tested using WST-1 assay. The compounds were tested for their ability to inhibit the infection of DENV2, ZIKV, KUNV, and TBEV, and the most active compounds were also analyzed using the replicon-based assay. Interactions of one of the identified inhibitors with possible viral targets were studied using molecular dynamics simulations. **Results.** Two out of eight previously reported DENV2/DENV4 inhibitors demonstrated the ability to inhibit all studied viruses at low micromolar concentrations. Compound **C6** demonstrated the ability to inhibit both DENV2 and TBEV. Compounds **C1** (lycorine), **C3** (mycophenolic acid), and **C7** (vidarabine) were demonstrated as inhibitors of TBEV infection for the first time. **Conclusions.** Several compounds, previously described as inhibitors of DENV, are also able to inhibit other flaviviruses. This work is the first report on the anti-TBEV activity of lycorine (**C1**) and mycophenolic acid (**C3**), as well as vidarabine (**C7**). In addition, this is the first experimental confirmation of the antiviral activity of compound **C5** and the lack of detectable antiviral activity of compound **C8,** demonstrating the necessity of experimental verification of the computational predictions.

## 1. Introduction

Flaviviruses (genus *Flavivirus*) are the most common arthropod-borne viruses worldwide, and nearly 50% of all identified flaviviruses are human pathogens causing illness in millions of people worldwide (up to 400 million human infections annually) [1,2,3]. Most flaviviruses are transmitted to humans by mosquito or tick bites [4]. Flaviviruses are responsible for different illnesses in humans such as encephalitis [5], hemorrhagic fever [6], Guillain–Barré Syndrome [7], congenital malformations, and microcephaly [8].

Flaviviruses are positive-stranded RNA viruses with a genome size of approximately 11 kb. The viral genome encodes for structural and non-structural proteins, translated from a single open reading frame as a single polyprotein in the cell cytoplasm. This polyprotein is cleaved by viral and host proteases, releasing three structural proteins: capsid (C); membrane (M), which is expressed as the precursor (prM); envelope (E) proteins, as well as seven non-structural (NS) proteins, namely, NS1, NS2A, NS2B, NS3, NS4A, NS4B, and NS5. All non-structural proteins are involved in viral RNA replication, while structural proteins are responsible for forming virions [9].

Currently, efficient vaccines exist against a few flaviviruses: yellow fever virus (YFV), Japanese encephalitis virus (JEV) [10], and tick-borne encephalitis virus (TBEV) [11]. Despite the existence of effective vaccines, the YFV is still responsible for most cases of hemorrhagic fever and related mortality (up to 50%) worldwide [12]. In 2015, the DENV vaccine was approved in several countries [13]. However, this vaccine was not effective for children younger than 9 years old; therefore, later, in 2017, the vaccine was withdrawn from the market due to safety issues [14]. The limited number of flavivirus vaccines, as well as the lack of commercially available antivirals for flaviviral infections, makes symptomatic relief a basic treatment option [15]. The rapid increase and expansion of flaviviral infections and the lack of effective therapies emphasize the importance of finding effective antivirals to treat such diseases.

Antiviral agents can be divided into two groups: (I) direct-acting antivirals and (II) host-targeting antivirals [16]. The development of directly acting antivirals is focused on the search for the specific small-molecule compounds directly affecting the important viral targets (viral proteins or genomic RNA). The main benefit of direct-acting antivirals is not only the reduction in adverse drug reactions but also the increase in sustained virologic response rates [17]. The activities of host-acting antivirals are based on the interference with any pathway important for different steps of the viral life cycle (binding to the cell receptor(s), entry, uncoating, replication, formation, and release of new viral progeny). This approach can be used to find broad-spectrum antiviral agents since multiple viruses often employ the same components of host cell machinery. In addition, host-targeting drugs allow avoiding the development of drug resistance [18]. In both approaches, one can highlight the search for broad-spectrum flavivirus inhibitors separately. Shared transmission pathways, the epidemiological and ecological features, and the ability to co-infect the same host make this approach an attractive strategy for the search and development of effective drugs against flavivirus infections [19,20]. Several attempts have been made to find potential broad-spectrum antivirals effective against multiple flaviviruses. For example, Thames et al. reported a novel flexible nucleoside analogue with moderate activity against DENV and YFV potentially targeting the GTP binding site of the NS5 [21]. Recently, two compounds targeting NS5 methyltransferase with activity in the low micromolar range against DENV3 and ZIKV were reported by Samrat et al. [22]. Also, several most recent publications give a detailed overview of the current achievements in the development of broad-spectrum flaviviral inhibitors as well as the perspectives of this approach [19,20,23].

One of the most essential steps in the development of new antivirals targeting specific viruses as well as broad-spectrum antivirals is the identification and selection of the molecular target [24]. The potential molecular target for broad-spectrum drugs should have a crucial role in the viral life cycle and should have a low rate of non-lethal, but resistance-conferring mutations [19]. In flaviviruses, the NS2B/NS3 protease is a promising drug target, which satisfies these conditions. The flaviviral NS3 is a key multifunctional viral protein that acts as a 5′ RNA triphosphatase, a nucleoside triphosphatase, a helicase, and a serine protease (in cooperation with NS2B) [22,25]. The function of NS3 as a protease has a crucial role in virus replication [26]. The sequence alignment for NS3 of different flaviviruses shows the sequence identity among JEV, West Nile virus (WNV), DENV, YFV, and Zika virus (ZIKV) from 50% up to 75% [27]. The other promising drug target for the development of broad-spectrum inhibitors is flaviviral NS5 polymerase [19]. NS5 (a catalytic module of the replication complex) is the largest flaviviral protein and has a fundamental role in flavivirus replication [25]. Similar to the NS3 protease, the NS5 polymerase is one of the most conserved of the flavivirus proteins [28,29]. According to the alignment of flaviviral NS5 proteins, the sequence identity is higher than 50% for KUNV, ZIKV, and TBEV, but DENV seems to be different, although the GDD catalytic triad is conserved in all flaviviruses (Figure S1).

In this work, we focused on the search for the possible broad-spectrum antiviral agents with activity against ZIKV, Kunjin (KUNV, Australian strain of WNV), and TBEV among the previously reported DENV type 2 (DENV2) or type 4 (DENV4) inhibitors potentially targeting either the NS2B/NS3 helicase/protease or NS5 RNA-dependent RNA polymerase (RdRp) of flaviviruses.

**Figure 1:**
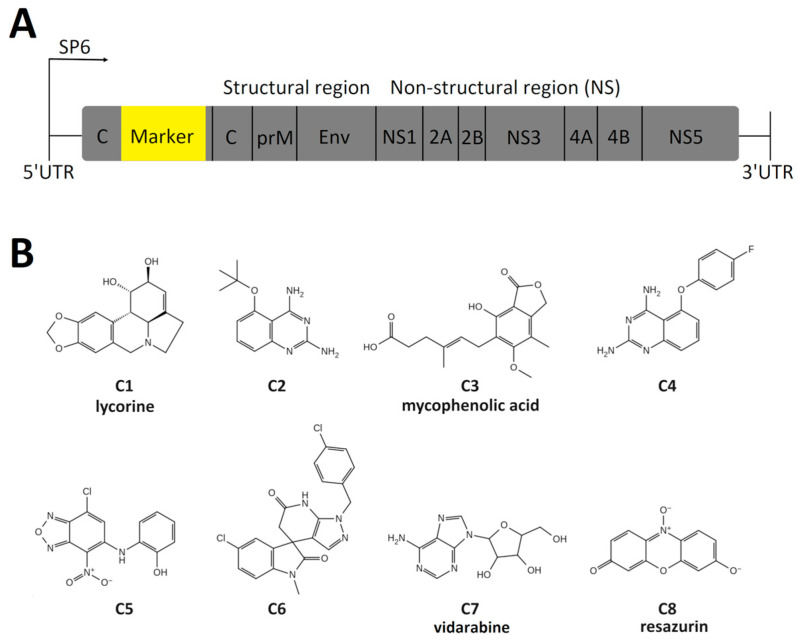
(**A**) The schematic picture of the genome of flaviviruses with NanoLuc reporter. “Marker” contains NanoLuc and FMDV 2A auto protease for cleavage of the reporter protein from the viral polyprotein. (**B**) The 2D structures of the selected compounds.

For rapid, high-throughput assays, genetically modified viruses expressing reporter proteins, such as luciferases or fluorescent proteins, are often used, as they allow for easy and rapid quantitation/visualization of viral replication/infection [30,31]. In the current work, we used flaviviruses with the Nanoluc reporter inserted in frame between the duplicated viral capsid encoded sequence (Figure 1A).

## 2. Results

### 2.1. Selection of the Compounds

To identify potential candidates for the testing as the potential broad-spectrum flavivirus inhibitors, the 80 compounds with previously reported activity against the DENV, DENV2, or DENV4 (target ChEMBL IDs: CHEMBL613757, CHEMBL613966, CHEMBL613728, CHEMBL5980, respectively) with EC_50_ equal to or lower than 10 μM were selected from the ChEMBL database [32] (Table S1). Thereafter, eight compounds were randomly selected from the initial dataset for further testing (Figure 1B). The initially selected compounds that were not commercially available were replaced by analogues with a Tanimoto coefficient higher than or equal to 0.7 [33].

### 2.2. Cytotoxic Effect of the Studied Compounds on Vero E6 Cells

**Figure 2:**
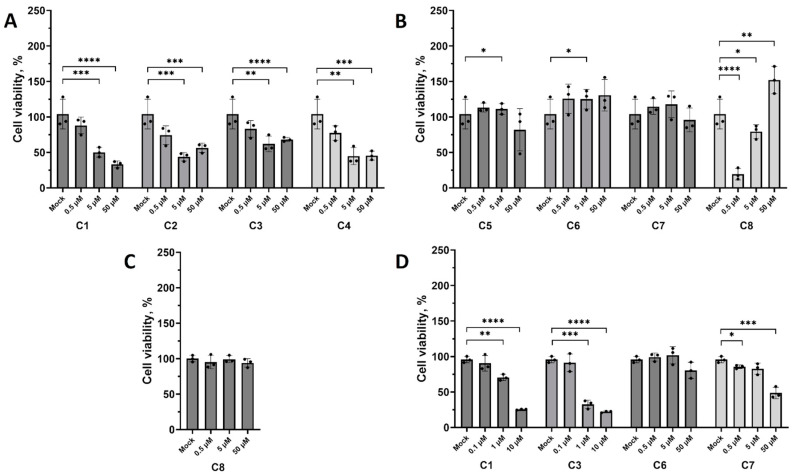
(**A**,**B**) Viability of Vero E6 cells treated with studied compounds, assessed by WST-1 assay after 48 h of exposure. (**C**) Viability of Vero E6 cells treated with compound **C8**, assessed by resazurin cell viability assay after 48 h of exposure. (**D**) Viability of U2OS cells treated with the selected compounds, assessed by WST-1 assay after 48 h of exposure. The cells treated with 0.01% of DMSO were used as a solvent control. Viability of cells treated with 0.01% of DMSO was taken as 100%. Data are presented as mean ± SD of 3 independent measurements. * *p* < 0.05, ** *p* < 0.01, *** *p* < 0.001, **** *p* < 0.0001, one-way ANOVA test.

The cytotoxic effect of all studied compounds was assessed using the WST-1 assay after 48 h of exposure. Compounds **C1**–**C4** were extremely toxic at concentrations higher than 0.5 μM (Figure 2A,B). Compound **C5** had a moderate cytotoxic effect at 50 μM, while compounds **C6** and **C7** had no cytotoxic effect at the maximal tested concentration (Figure 2A). Compound **C8** demonstrated the inverse dose-dependent effect in this assay. This observation can be explained by the fact that the compound **C8** is resazurin, phenoxazine dye, also known as Alamar Blue, which is subject to irreversible metabolic reduction to the resorufin by live mammalian cells [34]. Thus, the cytotoxic effect of this compound cannot be assessed by the WST-1 assay correctly. Therefore, the **C8** cytotoxicity was further assessed using the resazurin cell viability assay. This assay allowed us to avoid the interference between the tested compound and dye and obtain more reliable results for colored compounds. According to the results, the **C8** had no cytotoxic effect on Vero E6 after 48 h of exposure (Figure 2C).

Later, the cytotoxic effect of the compounds most active against the studied viruses was also determined on the human osteosarcoma U2OS cell line. As can be seen in Figure 2D, the cytotoxic effect of **C1** and **C6** on the U2OS cell line is similar to their effect on the Vero E6 cell line. Compound **C7** turned out to be more toxic for the U2OS cells at the maximum tested concentration than in the Vero E6 cells. Compound **C3**, in turn, was extremely toxic for the U2OS cell line even at the lowest micromolar concentration. The previously reported 50% cytotoxic concentration (CC_50_) of **C3** (mycophenolic acid) against U2OS cells is 1.66 μM [35], which is in line with our results.

### 2.3. Verification of the Activity of the Selected Compounds Against DENV

**Figure 3:**
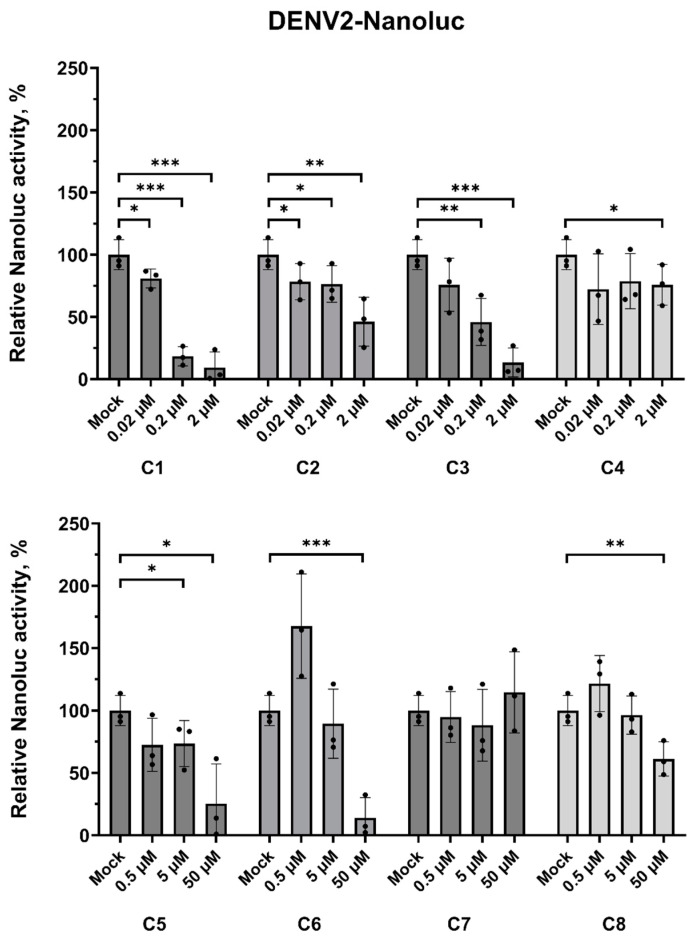
The antiviral effect of the studied compounds against DENV2-Nanoluc, 24 hpi; The cells treated with 0.01% of DMSO were used as a solvent control (Mock). Nanoluciferase activity in infected cells treated with 0.01% of DMSO was taken as 100%. Data presented as mean ± SD of 3 independent experiments: * *p* < 0.05, ** *p* < 0.01, *** *p* < 0.001, one-way ANOVA test.

The next step of this study was focused on verifying the activity of the eight randomly selected compounds against DENV2 since some of the initially selected compounds with previously reported activity against DENV2 or DENV4 were replaced by their structural analogues (Table S1). The antiviral effect of the compounds was assessed on Vero E6 cells infected at a multiplicity of infection (MOI) of 0.1 using recombinant DENV2 with nanoluciferase (DENV2-Nanoluc) reporter at 24 h post-infection (hpi). The tested concentration range for each compound was selected based on the WST-1 cell viability assay results. Compounds **C1**–**C4** were tested at concentrations ranging from 0.02 μM up to 2 μM with a ten-fold dilution step, and compounds **C5**–**C8** were tested at concentrations ranging from 0.5 μM up to 50 μM with a ten-fold dilution step. The initial screening of the antiviral activity showed that only five out of eight compounds were able to inhibit DENV2-Nanoluc activity with apparent dose-dependent antiviral effects (Figure 3). These compounds (**C1**, **C2**, **C3**, **C5**, and **C6**) were selected for further half-maximal effective concentration (EC_50_) determination. In addition, some negligible inhibition effect at the maximum tested concentration was obtained for compounds **C4** and **C8**. In the case of compound **C4**, this negligible antiviral effect can be related to the cytotoxicity of the compound.

### 2.4. Evaluation of the Antiviral Activity of Compounds Against ZIKV, KUNV, and TBEV

All eight compounds were studied for their ability to inhibit the activity of the recombinant ZIKV, KUNV, or TBEV with Nanoluc reporter in Vero E6 cells infected at an MOI of 0.1 at 24 hpi (KUNV and TBEV) or 32 hpi (ZIKV). The antiviral activity of the compounds was tested using the same concentration range as in the case of the DENV2-Nanoluc. According to the initial screening results, five out of eight compounds demonstrated a significant antiviral effect against all studied viruses.

**Figure 4:**
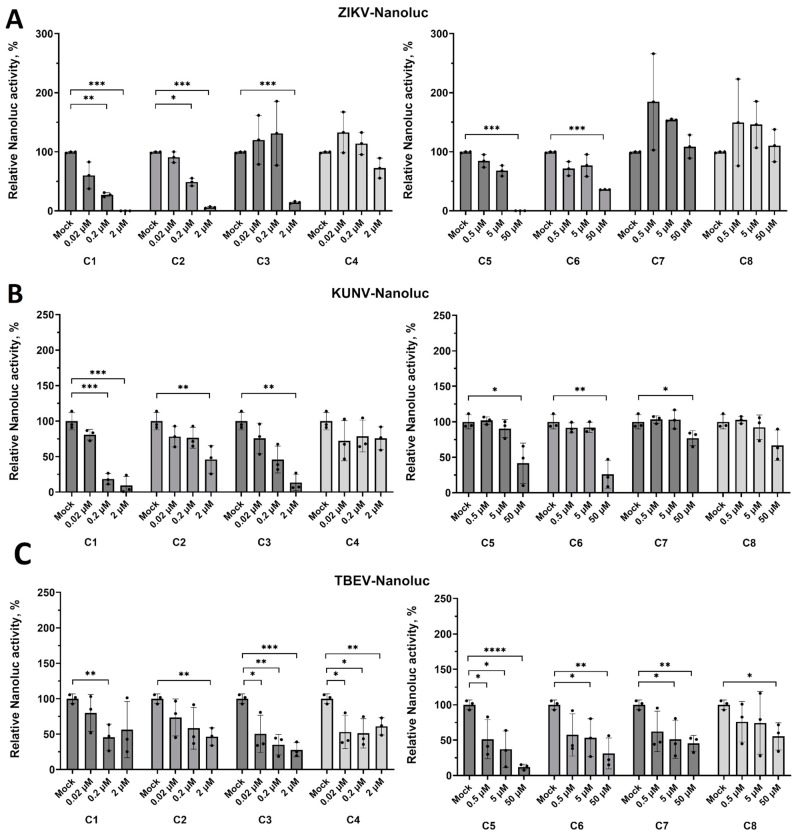
(**A**) The antiviral effect of the studied compounds against ZIKV-Nanoluc, 32 hpi. (**B**) The antiviral effect of the studied compounds against KUNV-Nanoluc, 24 hpi; (**C**) The antiviral effect of the studied compounds against TBEV-Nanoluc, 24 hpi. The cells treated with 0.01% of DMSO were used as a solvent control (Mock). Nanoluciferase activity in infected cells with 0.01% of DMSO was taken as 100%. Data presented as mean ± SD of 3 independent experiments: * *p* < 0.05, ** *p* < 0.01, *** *p* < 0.001, **** *p* <0.0001, one-way ANOVA test.

In the case of the ZIKV-Nanoluc, five compounds were able to inhibit its activity in a dose-dependent manner (Figure 4A). A statistically significant antiviral effect of compounds **C1** and **C2** appeared at the 0.2 μM concentration, and both compounds completely inhibited the ZIKV-Nanoluc replication at the maximum tested concentration (Figure 4A). Compound **C3** only demonstrated a statistically significant antiviral effect at the maximum tested concentration (Figure 4A). Compounds **C5** and **C6** can be suggested as moderate ZIKV inhibitors (Figure 4A). However, it can be assumed that **C5** is a more potent ZIKV inhibitor since, at the 50 μM concentration, the virus activity was completely inhibited. In contrast, **C6** partially inhibited the virus activity at this concentration (Figure 4A). As in the case of DENV2-Nanoluc, compound **C4** demonstrated some tendency to decrease the ZIKV-Nanoluc activity at a concentration of 2 μM, which can also result from the cytotoxic effect of the compound on Vero E6 cells.

In the case of the KUNV-Nanoluc virus, the apparent dose-dependent antiviral effect was obtained only for compounds **C1** and **C3**, while compounds **C2**, **C5**, and **C6** decreased the KUNV-Nanoluc activity only at the maximum tested concentrations (Figure 4B). In addition, a slight inhibition effect was observed for compound **C7** at the 50 μM concentration.

In the case of TBEV-Nanoluc, a clear dose-dependent antiviral effect was obtained for compounds **C2**, **C3**, **C5, C6,** and **C7** (Figure 4C). The antiviral effect of compound **C1** appeared at a concentration of 0.2 μM; interestingly, the ten-fold increase in concentration did not lead to any significant improvement of the antiviral effect (Figure 4C). Similar results were obtained for compounds **C4** and **C8**. Both compounds demonstrated the ability to partially inhibit viral replication at the minimal tested concentration without an apparent dose-dependent effect.

Based on the initial screening of antiviral activity, compounds **C1**, **C2**, **C3**, **C5,** and **C6** were selected to further determine the EC_50_ and CC_50_, as well as to identify possible protein targets and theoretically study the possible mechanism of action.

### 2.5. Determination of the CC_50_, EC_50_, and Selectivity Index of the Selected Compounds

The identified compounds with activity against all studied flaviviruses were tested in the broader concentration range selected according to the cytotoxicity of the compound: **C1**, **C2**, and **C3** were tested at concentrations ranging from 0.031 μM up to 2 μM with a two-fold dilution step, and compounds **C5** and **C6** were tested at concentrations ranging from 0.78 μM up to 100 μM with a two-fold dilution step. It was found that the EC_50_ of **C1**, **C2**, and **C3** against all studied viruses is in the sub-micromolar range, while the EC_50_ of **C5** and **C6** is in the micromolar range (Table 1), which aligns with the preliminary data.

As compounds **C1**, **C2**, and **C3** were relatively toxic (Figure 2A), we determined the selectivity index (SI) of the identified potential broad-spectrum flavivirus inhibitors. As the WST-1 assay tends to underestimate the toxic effect and is unable to detect cytostatic effects, the CC_50_ of the perspective compounds was measured using a more sensitive xCELLigence real-time cytotoxicity assay (RTCA). The CC_50_ of compounds in the Vero E6 cell line was calculated at 24 h of exposure for all compounds according to the time of the most viral infection experiments. The full xCELLigence RTCA data are provided in the Supporting Information section (Figures S2 and S3). Thereafter, for each compound, the SI was calculated as a ratio of the CC_50_ of a compound against its EC_50_ [36].

**Table 1:** The 50% cytotoxic concentration (CC_50_) and selectivity index (SI) of the most active compounds against all studied viruses. Doxorubicin (for DENV2) and Arbidol (for ZIKV, KUNV, and TBEV) are used as reference inhibitor compounds.

Compound	CC_50_, μM Vero E6	DENV2		ZIKV		KUNV		TBEV	
		EC_50_, μM	SI	EC_50_, μM	SI	EC_50_, μM	SI	EC_50_, μM	SI
Doxorubicin [37]	24.0	1.2	20	-	-	-	-	-	-
Arbidol [38]	89.7	-	-	12.09	7.4	18.78	4.8	18.67	4.8
**C1**	1.3	0.09	14.4	0.13	13.0	0.17	7.6	0.09	14.0
**C2**	0.31	0.13	3.4	0.48	0.6	0.26	1.2	ND **	-
**C3**	>5	0.12	>40	0.53	>9	0.21	>23	0.61	>8
**C4**	>0.2	NA *	-	NA	-	NA	-	NA	-
**C5**	12.8	2.91	4.4	10.41	1.2	8.90	1.4	9.88	1.3
**C6**	>50	3.8	>10	23.93	2.1	20.58	2.4	13.95	3.6
**C7**	>50	NA	-	NA	-	NA	-	8.33	6.0
**C8**	>50	NA	-	NA	-	NA	-	NA	-

* NA—not active; ** ND—not determined.

### 2.6. Evaluation of the Antiviral Activity of Compounds Using Replicon-Based Assay

To study the effect of the identified potential broad-spectrum flavivirus inhibitors (**C1**, **C3**, **C6**) and novel potential TBEV inhibitor **C7** on viral replication, we further tested their activity using replicon systems of studied flaviviruses. The compounds were added at different time points: 1 h and 8 h post-transfection. The tested concentrations for all compounds were selected based on their cytotoxicity in U2OS cells determined using WST-1 assay after 48 h of exposure.

**Figure 5:**
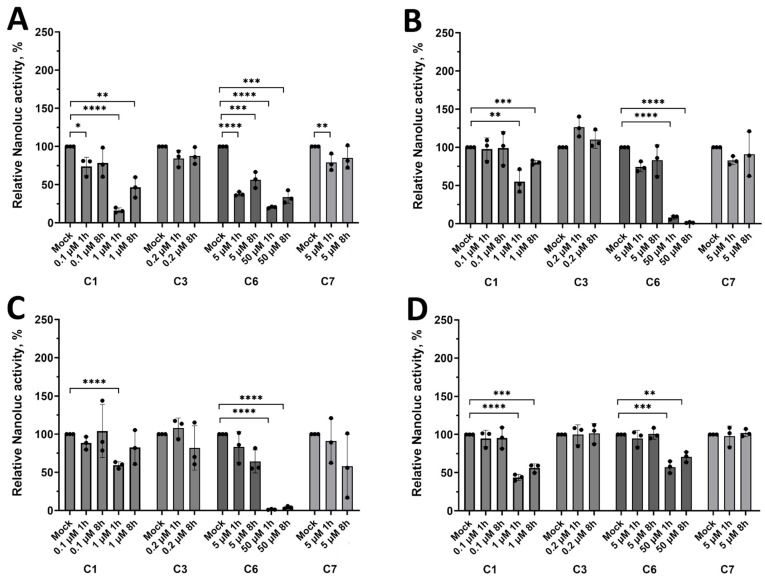
The antiviral effect of the studied compounds against (**A**) DENV2-Nanoluc, (**B**) ZIKV-Nanoluc, (**C**) KUNV-Nanoluc, and (**D**) TBEV-Nanoluc replicons. The cells treated with 0.05% of DMSO were used as a solvent control (Mock). Nanoluciferase activity in cells treated with 0.05% of DMSO was taken as 100%. Data presented as mean ± SD of 3 independent measurements: * *p* < 0.05, ** *p* < 0.01, *** *p* < 0.001, **** *p* < 0.0001, one-way ANOVA test.

Interestingly, in the replicon assays, compound **C6** was the most active against all the studied viral replicons indicating inhibition or viral RNA translation and/or RNA replication. In contrast, compound **C3**, suggested to be a potential broad-spectrum flavivirus inhibitor based on the experiments with recombinant viruses with Nanoluc reporter, had no effect at the maximum non-toxic concentration (Figure 5), suggesting that its antiviral activity is due to inhibiting other stage(s) of the viral life cycle. For compound **C1**, the obtained results were in line with the suggestion that the possible target for this compound in flaviviruses is the NS4B-2K protein, which has an important role in viral RNA replication. The lower inhibitory effect of the compound in the replicon assays may be related to the fact that these assays cover only viral translation and replication steps but do not include aspects of viral entry, virion assembly/release, and spread in the cell culture. With all four virus replicons, it could be assessed that later addition (at 8 h post-transfection) of **C1** had a lower antiviral effect compared to earlier (at 1 h post-transfection), which could be expected from compound-inhibiting viral RNA replication (Figure 5). Compound **C7**, a novel potential TBEV inhibitor, also had a slight inhibition effect against DENV2-Nanoluc if it was added at 1 h post-transfection, but no inhibition of TBEV replication was observed regardless of whether it was added at 1 h or 8 h post-transfection. The results suggest that the compound **C7** does not affect the viral RNA translation and/or replication.

### 2.7. Identification of the Possible Molecular Targets and Molecular Dynamics (MD) Study of the Interactions Between Most Active Compounds and Their Molecular Targets

To investigate the possible mechanism of action of the most attractive candidates identified based on SI, compounds **C1** and **C3**, we attempted to identify their possible molecular targets in the studied flaviviruses. Based on the literature search, it was found that the possible target for **C1** in flaviviruses is the 2K–NS4B polyprotein [39]. Since there is no experimentally determined 3D structure of the flaviviral NS4B [40] and due to the highly dynamic nature of this protein [41], the MD study of the interactions between **C1** and NS4B was not carried out. It was previously shown that compound **C3** inhibits flavivirus infection by preventing the synthesis and accumulation of viral RNA [42]. Thus, the NS3 protease/helicase and RNA-dependent RNA polymerase (RdRp) domain of NS5 can be suggested as potential molecular targets for this compound.

Before the MD simulation, the binding poses of **C3** to the active site of NS2B/NS3 and the allosteric pocket of the NS5 of the studied flaviviruses were calculated using AutoDock Vina 1.1.2 [43]. The calculated binding energies, ligand efficiencies (LE), and interaction between compound **C3** and NS2B/NS3 and NS5 proteins of the studied viruses are given in Table 2. In the case of NS2B/NS3, compound **C3** potentially binds near the catalytic triad of NS3 (Figure S4). For NS5 RdRp, the potential binding site for compound **C3** is the region near the priming loop (Figure S5). As can be seen from Table 2, in the case of NS5, the RdRp compound **C3** forms more specific interactions, i.e., hydrogen bonds, than with NS2B/NS3. However, the calculated binding energies and LE are almost the same for NS2B/NS and NS5 RdRp, which does not allow us to suggest with which protein binding is preferable.

Thereafter, the MD simulations of 50 ns were carried out for each studied complex. The evaluation of the root mean square deviation (RMSD) of the compound **C3** and protein atom positions in time showed that the complexes of **C3** with DENV NS5 and ZIKV NS5 are more stable than its complexes with NS5 RdRp of TBEV or KUNV (WNV) (Figure S6).

**Figure 6:**
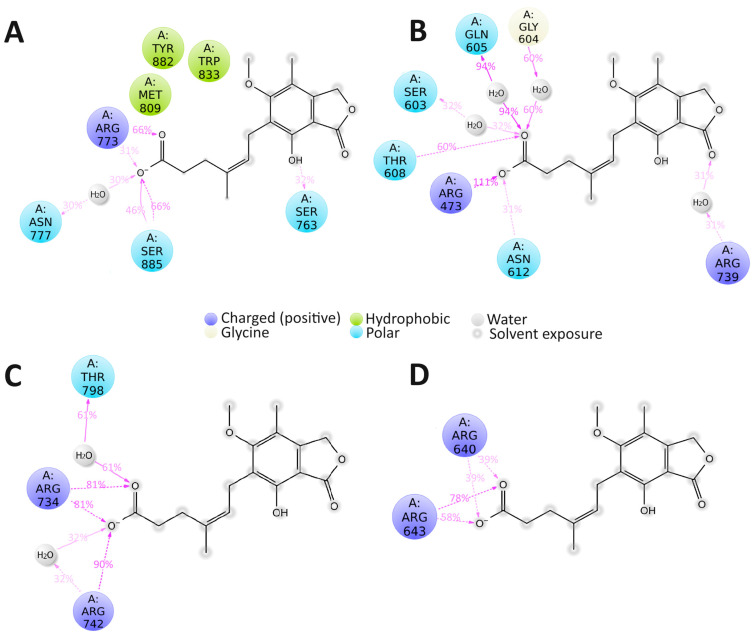
The 2D diagram of the MD calculated contacts for complexes of compound **C3** with (**A**) DENV2 NS5 (PDB ID: 6IZX), (**B**) ZIKV NS5 (PDB ID: 6LD1), (**C**) WNV NS5 (PDB ID: 2HCN), and (**D**) TBEV NS5 (PDB ID: 7D6N). The hydrogen bonds between the ligand and backbone of amino acid residue are shown as a purple solid arrow. The dashed purple arrows denote the hydrogen bond between the ligand and the side chain of amino acid residue. The contacts that occur over 30% of the simulation time are shown.

**Figure 7:**
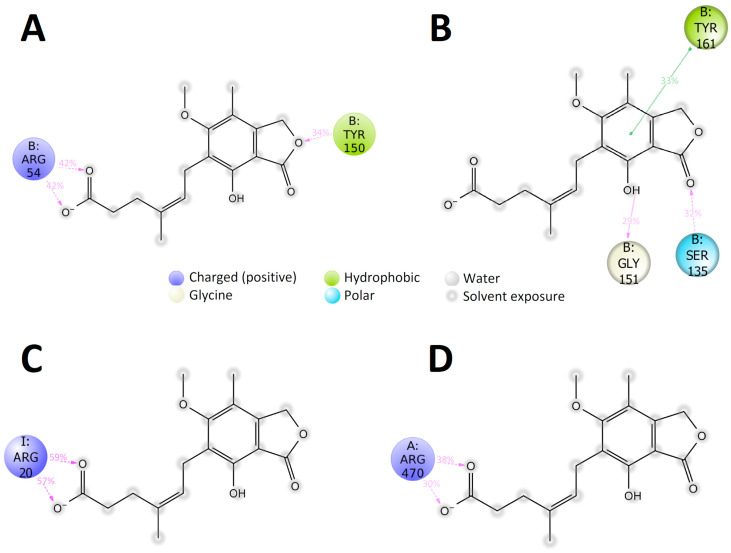
The 2D diagram of the MD calculated contacts for complexes of compound **C3** with (**A**) DENV2 NS3 (PDB ID: 2FOM), (**B**) ZIKV NS3 (PDB ID: 7OBV), (**C**) WNV NS3 (PDB ID: 2IJO), and (**D**) TBEV NS3 (full-length structure generated by SWISS-MODEL (https://swissmodel.expasy.org/ accessed on 16 February 2025)). The hydrogen bonds between the ligand and backbone of amino acid residue are shown as a purple solid arrow. The dashed purple arrows denote the hydrogen bond between the ligand and the side chain of amino acid residue. The contacts that occur over 30% of the simulation time are shown.

The same tendency was also observed for the complexes of **C3** with NS2B/NS3. The complex of **C3** with NS2B/NS3 of ZIKV was stable, while the ligand RMSD value was very high for other complexes (Figure S7). Such large ligand RMSD values can indicate that ligand binding with the active site of the NS2B/NS3 or with the allosteric pocket of NS5 is unstable and that the ligand diffused away from its initial binding site during the MD simulation. The stability of the MD simulation results was confirmed by additional MD runs of 25 ns for each complex (data not shown). In all cases, the most stable, long-maintained, over the simulation time, specific contacts were formed with the participation of the compound carboxyl group. Namely, in the complex NS5 DENV2–**C3**, the carboxyl group participated in the forming of the hydrogen bonding between side chains of Ser885 and Ser763 amino acid residues and water bridge with sidechain of Asn777 (Figure 6A). In the case of ZIKV, the carboxyl group of **C3** contributed to the forming of six contacts occurring over 30% of the simulation time: water bridges with a backbone of Gly604 and Gln605, and side chains of Arg473, Ser603, and Arg739, and two hydrogen bonds with side chains of Arg473 and Asn612 (Figure 6B). In the case of WNV (KUNV) and TBEV NS5 polymerases, over the simulation time, the number of long-maintained, specific interactions was significantly lower than in the case of DENV2 and ZIKV. Compound **C3** formed two hydrogen bonds with side chains of Arg734 and Arg742 and a water bridge with a backbone of Thr798 amino acid residue of WNV NS5 (Figure 6C). Only two long-maintained hydrogen bonds were observed in the TBEV NS5–**C3** complex; both were formed with the participation of the carboxyl group of the compound and side chains of Arg640 and Arg643 of TBEV NS5 (Figure 6D). In the case of NS2B/NS3, over the simulation time, the number of long-maintained, specific contacts was dramatically lower than in the case of NS5 (Figure 7). Compound **C3** formed only a few contacts with the NS3 protease domain of all studied viruses.

## 3. Discussion

The previously reported DENV2 and DENV4 inhibitors were employed as starting compounds to identify the potential broad-spectrum inhibitors of flaviviruses. Here, we demonstrated that two out of eight compounds, **C1** and **C3** can in vitro inhibit the infection of DENV2, ZIKV, KUNV, and TBEV. As can be seen from Table 1, both compounds **C1** and **C3** have an SI higher than three for all the studied viruses; therefore, they can be considered as potential candidates for further development of broad-spectrum flavivirus inhibitors. **C1** and **C3** are known and well-described compounds. However, our work is the first demonstration of the antiviral activity of both of these compounds against tick-transmitted TBEV. For **C1**, our results are in line with the previously published data. Previously, compound **C1**—lycorine, a natural plant alkaloid—was reported to reduce viral titers of WNV, DENV, and YFV by 10^2^- to 10^4^-fold at the concentration of 1.2 μM [44] and inhibit ZIKV replication in Vero E6 cells with an EC_50_ of 0.39 μM [45]. In our experiments, lycorine inhibited the ZIKV-Nanoluc produced reporter activity with an EC_50_ of 0.13 μM (Table 1).

The compound **C3**—mycophenolic acid, an approved immunosuppressant [46,47]—was more active against DENV2 than it was previously reported. Chu et al. showed that **C3** inhibits DENV-2-NGC activity in Vero E6 cells with an EC_50_ of 5.7 μM [48]. In the case of ZIKV, our results for **C3** are consistent with published data. According to Barrows et al., the compound can inhibit ZIKV MEX_I_7 activity in different human cells with an EC_50_ between 0.1 and 1 μM [49]. It is worth noting that mycophenolic acid (compound **C3**) was previously suggested as a potential inhibitor with broad antiviral properties against flaviviruses [19]. In addition, two other compounds were found to be of potential interest for further development of new antivirals. The ability of compound **C6** to inhibit the activity of DENV2 and TBEV with an EC_50_ lower than 15 μM and the very low cytotoxicity (CC_50_ > 50 μM) of the compound allows us to consider it as a potential candidate (scaffold) for the further development of dual inhibitors. Based on the experimentally obtained values of EC_50_ and SI—8.33 μM and 6, respectively—compound **C7** can also be selected for further development as a novel TBEV inhibitor. It should be noted that compound **C7** is a well-described antiviral drug, vidarabine, active against *herpes simplex* and *varicella zoster* viruses [50]; here, for the first time, we report on the anti-TBEV activity of this compound.

Since all studied compounds are supposed to be direct-acting antivirals, the analysis of the potential viral protein targets was performed. According to the published data, the potential protein target in flaviviruses for compounds **C1** and **C6** is a membrane protein NS4B [28,39,44,51]. The study of possible interactions between these compounds and NS4B is complex and most likely not informative due to the highly dynamic nature of this protein and the absence of the experimentally determined structure [40,41]. However, using a replicon-based assay, we demonstrated that both compounds can inhibit the RNA translation/replication stages of viral infection. According to these results, compound **C6** is more active than compound **C1**, while in experiments with recombinant viruses, **C1** is more active. It can be suggested that the effect of compound **C1** is probably related not only to the inhibition of the virus replication/translation but also to the viral entry or virion assembly/release and virus spread in cell culture, which are not covered in the replicon-based assays. Compound **C6**, as the most active compound in replicon-based assays, clearly causes inhibition of viral RNA replication. Based on its activity, it can be suggested that this is the main or only mechanism of action of this compound. For compound **C3**, the NS5 RdRp and NS2B/NS3 helicases were identified as possible targets. Based on the analysis of the stability of the complexes of **C3** with NS2B/NS3 or NS5 RdRp, it can be suggested that the binding of the compound with the NS5 RdRp domain is preferable to that with NS2B/NS3 protease/helicase, which is in line with the previously published data [52]. In the replicon-based assays, however, the compound **C3** was not active at the maximum non-toxic concentration. Unfortunately, the high toxicity of the compound (Figure 2D) in U2OS cells did not allow us to make a conclusion on the possible mechanism(s) of its action. According to the molecular docking and MD simulations results, in all cases, the compound **C3** was bound to the NS5 near the priming loop, which positions nucleotides for polymerization [53]. This localization of the compound probably prevents conformational changes required for the elongation of the RNA. The analysis of the MD-calculated interactions between the compound **C3** and NS5 proteins of all studied viruses showed that the carboxyl group of the compound **C3** can play a central role in the binding with NS5. Obviously, the immunosuppressive properties of mycophenolic acid, compound **C3**, serve as a major obstacle to the use of this compound as a potential broad-spectrum antiviral agent. However, the combination of the in vitro and in silico techniques gives valuable insight into the potential mechanism of action of the compound at the atomic level, which can greatly impact the further development of the **C3**-based broad-spectrum inhibitors.

It can be suggested that compounds **C2** and **C4** have the same molecular target in the flaviviruses since these compounds are structural analogues. Both compounds contain 2,4-diaminoquinazoline cores, and these types of compounds have been previously reported as potential inhibitors of flavivirus replication [54]. Since the flavivirus replication process is related to the activity of the different non-structural proteins, identifying the potential molecular target of 2,4-diaminoquinazoline derivatives requires extensive additional studies. Taking into account the fact that compound **C5** was active against all studied viruses, NS2B/NS3 helicase/protease or NS5 RdRp, as the most conservative proteins in flaviviruses, can be suggested as potential molecular targets for this compound [28,29]. However, it should be noted that the observed antiviral activity of compound **C5** can also be related to the high cytotoxicity of this compound, which in turn is most likely related to the toxicity issues of the nitro group [55]. Compound **C7** is previously known as an antiviral drug against different DNA-genome viruses interfering with the synthesis of viral DNA [56]. In replicon-based assays, the compound was inactive against all studied flavivirus replicons at the maximal non-toxic concentration. Based on these results, it can be suggested that **C7** does not affect the viral RNA replication and translation processes but possibly inhibits the late stages of viral infection. The potential mechanism of its action as well as a molecular target identification in flaviviruses requires additional study.

As an additional and important result of the work, we found that the previously published data on biological activity should be verified in some cases. For compound **C2**, our results contradict the previously published data. Saul et al. showed that compound **C2** (Compound **6** in the original publication) has an EC_50_ of 2.8 nM against the New Guinea C strain of DENV2; in this case, human Huh7 cells were infected for 48 h and the antiviral effect was measured using the *Renilla* luciferase assay [57]. Similar results were also reported by Chao et al. (Compound **4o** in the original publication) [54]. However, in our experiments, the EC_50_ of **C2** against DENV2 was around 130 nM (0.13 μM; Table 1); the difference may be due to the use of different cell types, virus strains, or assay conditions. More strikingly, Zou et al. reported that compound **C6** inhibits DENV2 infection in BHK-21 cells with an EC_50_ of 6 nM [51]; meanwhile, here, we observed that the EC_50_ of **C6** against DENV2 in Vero E6 cells was approximately 600-fold higher (3.8 μM; Table 1). Such a significant difference is difficult to explain by the different methods used to evaluate the antiviral activity of the compound.

In the case of compounds **C5** and **C8**, no published experimental data on their antiviral activity exist. Both compounds were predicted to be potential DENV inhibitors with an EC_50_ of 9.7 μM and 1.6 μM, respectively, by Primary and Confirmatory Screening for Flavivirus Genomic Capping Enzyme Inhibition (PUBCHEM_BIOASSAY, AID588708, AID588742). In our experiments, **C5** was able to inhibit the DENV2-Nanoluc activity in Vero E6 cells with an EC_50_ of 2.91 μM (Table 1). Thus, it can be concluded that our results are the first experimental confirmation of the antiviral activity of **C5**. In contrast, compound **C8** had no antiviral effect against DENV2-Nanoluc in the tested concentrations range (Figure 3), which are also the first experimental data on the lack of detectable antiviral activity of this compound. In these cases, the differences between predicted and experimentally measured activities are expected. Despite modern developments, computational methods still remain theoretical and require experimental confirmation, and this work clearly demonstrated that the activities of all compounds, predicted in silico to act as inhibitors, should be experimentally verified. Compounds **C4** and **C7** are structural analogues (with minor substitutions) of the original compounds selected from the ChEMBL database [32]. The analogue of **C4** was previously described in the literature [54] as a potential DENV2 inhibitor with an EC_50_ of 60 nM obtained in experiments with the BHK-DENV-2-replicon system (Compound **4g** in the original publication). Unfortunately, both compounds turned out to be less active than reported for the original ones; in these cases, the lower efficacy may be related to the structural differences of compounds. This observation demonstrates that even a small substitution (fluorine atom in the para-position of the phenoxy group at the eighth position of 2,4-diaminoquinazoline) in the pharmacophore core of the lead-compound can lead to the total loss of antiviral activity. In addition, these results further demonstrate that the use of different methods and their combinations can significantly impact the efficient identification and development of new drug candidates.

## 4. Conclusions

In this work, based on the previously reported DENV2/DENV4 inhibitors, we proposed two potential broad-spectrum flavivirus inhibitors: one potential dual inhibitor of DENV2 and TBEV and a novel potential inhibitor of TBEV. Both potential broad-spectrum inhibitors are well-known and studied antivirals; however, this is the first report on the anti-TBEV activity of lycorine (**C1**) and mycophenolic acid (**C3**). This work is the first experimental confirmation of the antiviral activity of compound **C5** and also the first confirmation of the lack of detectable antiviral activity of compound **C8**. In addition, the potential molecular targets in flaviviruses were suggested for all identified compounds. The MD simulation study results and replicon-based assay allowed us to suggest that the flaviviral NS5 RdRp is a potential target for compound **C3**, and 2K-NS4B for compound **C1**. The obtained experimental results demonstrated the importance of verifying the previously published data on the biological activity and necessity of experimental verification of the activity of the computationally predicted novel active compounds.

## 5. Materials and Methods

### 5.1. Compound Library Design

The 80 compounds with previously reported activity against DENV2 or DENV4 with EC_50_ equal to or lower than 10 μM were selected from the ChEMBL database [32] (Table S1). Thereafter, 8 compounds were randomly selected from the initial dataset for further testing. The initially selected compounds that were not commercially available in MolPort Inc. [58] were replaced by their analogues with the Tanimoto coefficient [59] greater or equal to 0.7.

### 5.2. Compounds

**C1:**  (1S,17S,18S,19S)-5,7-dioxa-12-azapentacyclo[10.6.1.0^2^,^10^.0^4^,^8^.0^15^,^19^]nonadeca-2(10),3,8,15-tetraene-17,18-diol, TargetMol Chemicals, Cat. No. T3324;
**C2:**  5-(tert-butoxy)quinazoline-2,4-diamine, TargetMol Chemicals, Cat. No. T4197;
**C3:**  6-(4-hydroxy-6-methoxy-7-methyl-3-oxo-1,3-dihydro-2-benzofuran-5-yl)-4-methylhex-4-enoic acid, Apexbio Technology LLC, Cat. No. B1981;
**C4:**  5-(4-fluorophenoxy)quinazoline-2,4-diamine, Maybridge, Ltd., Cat. No. MWP01114;
**C5:**  2-[(7-chloro-4-nitro-2,1,3-benzoxadiazol-5-yl)amino]phenol, ChemBridge Corporation, Cat. No. 5241444;
**C6:**  5-chloro-1′-[(4-chlorophenyl)methyl]-1-methyl-1,1′,2,5′,6′,7′-hexahydrospiro[indole-3,4′-pyrazolo[3,4-b]pyridine]-2,6′-dione, INTERBIOSCREEN DOO BAR, Cat. No. STOCK6S-78535;
**C7:**  2-(6-amino-9H-purin-9-yl)-5-(hydroxymethyl)oxolane-3,4-diol, Apexbio Technology LLC, Cat. No. B2062;
**C8:**  sodium 3-oxo-3H-phenoxazin-10-ium-7,10-bis(olate), TargetMol Chemicals, Cat. No. T7650.

The 10 mM stock solutions were prepared by dissolving compounds in sterile dimethyl sulfoxide (DMSO) (Sigma, St. Louis, MO, USA) and stored at −20 °C until further use.

### 5.3. Cells

The African green monkey kidney epithelial clone cells E6 (Vero E6; ATCC CRL-1586) were grown in Dulbecco’s Modified Eagle’s Medium (DMEM) containing 10% of heat-inactivated fetal bovine serum (FBS) (PAN Biotech) and 100 IU penicillin/100 μg/mL streptomycin (Gibco, Grand Island, NY, USA). The cells were maintained at 37 °C in a humidified atmosphere with 5% CO_2_. Flp-In T-REx HEK 293 (Thermo Fisher Scientific, Waltham, MA, USA) cells were grown in DMEM containing 10% heat-inactivated FBS (Thermo Fisher Scientific, Waltham, MA, USA), 100 IU penicillin/100 μg/mL streptomycin, and 100 µg/mL Zeocin together with 15 µg/mL blasticidin; later, two antibiotics were omitted during virus rescue.

The human osteosarcoma cells U2OS (ATCC HTB-96) were maintained in Iscove Modified Dulbecco’s Medium (IMDM) containing 10% of heat-inactivated FBS (PAN Biotech, Aidenbach, Germany) at 37 °C in a humidified atmosphere with 5% CO_2_.

### 5.4. Viruses

The recombinant viruses with nanoluciferase (Nanoluc) reporters were used: (I) ZIKV-Nanoluc, a synthetic infectious clone constructed based on the BeH89015 sequence of the Brazilian strain [60,61]; (II) the recombinant DENV2-Nanoluc based on DENV2 strain 16681 (GenBank U87411.1); (III) the recombinant KUNV-Nanoluc based on the KUNV clone FLSDX (GenBank AY274504); (IV) the recombinant TBEV-Nanoluc based on the TBEV clone 93/783 (GenBank MT581212.1). Similarly to ZIKV, the Nanoluc (Nluc) was inserted into KUNV, DENV2, and TBEV infectious cDNAs cloned into the pCCI plasmid backbone as described before [62]. In the case of KUNV and TBEV, the marker was placed between duplicated copies of viral capsid protein (pCCI-KUNV-Nluc and pCCI-TBEV-93/783-Nluc) and, in the case of DENV2, between the truncated version and full-length sequence of capsid protein (pCCI-DENV2-CT-Nluc). The virus stocks were stored at −80 °C. All virus experiments (including virus rescue) were conducted in accordance with the guidelines of the national authorities using appropriate biosafety laboratories under all necessary safety approvals in the Core Facility for Biosafety (ABSL3) of the Institute of Technology University of Tartu (Ravila 14b, Tartu, Estonia).

### 5.5. Virus Rescue

In this work, the ZIKV-Nanoluc P1 and P2 working stocks were generated from the previously obtained ZIKV-Nanoluc P0 stock [61]. To generate P1 and P2 stocks, Vero E6 cells were infected with ZIKV-Nanoluc P0 or P1, respectively. Five days post-infection, the supernatants from ZIKV-Nanoluc-infected cells were collected, centrifuged at 1000× *g* for 5 min, and the aliquots were stored at −80 °C until further use. A CPER protocol was adopted to rescue viruses [63]. The DNA fragments covering the entire viral genome were amplified using Platinum SuperFi II DNA Polymerase (Thermo Fisher Scientific) (Table S2). For each virus, an additional linker fragment was also amplified; it contained a CMV-promoter, a hepatitis delta virus ribozyme (HDVr), and SV40 polyadenylation signal (pA) sequences. Further, all the fragments were mixed in equimolar amounts (0.1 pmol each) and subjected to CPER with Platinum SuperFi II DNA polymerase (2 min of denaturation at 98 °C; 20 cycles of 10 s at 98 °C, 15 s at 60 °C, and 12 min at 72 °C; final extension for 12 min at 72 °C). The crude unpurified CPER product mixture was directly transfected into Flp-In T-REx HEK 293 cells using Lipofectamine LTX (Thermo Fisher Scientific, Waltham, MA, USA), and P0 stock was harvested a week later. To obtain P1 working stock, Vero E6 cells were infected, and virus collection was carried out 5 days later.

### 5.6. WST-1 Cell Viability Assay

Vero E6 or U2OS cells were seeded on the 96-well plate at a density of 1 × 10^4^ cells/well and were allowed to adhere overnight. Next day, the cells were treated with the studied compounds at concentrations ranging from 1 to 100 μM with a ten-fold dilution step. As a solvent control, 0.01% DMSO (Sigma, St. Louis, MO, USA) was used. After 48 h of exposure, 10 μL of WST-1 (10 mg/mL) cell proliferation reagent (Roche, Cat. No. 11644807001) was added to each well, and plates were incubated at 37 °C, 5% CO_2_ for 2 h. The optical density was then measured at 450 nm using an Epoch Microplate Spectrophotometer (BioTek Instruments, Inc., Winooski, VT, USA). Four independent experiments were carried out in triplicates.

### 5.7. Resazurin Cell Viability Assay

Vero E6 cells were seeded on a 96-well plate at a density of 1 × 10^4^ cells/well and were allowed to adhere overnight. On the next day, the medium was replaced with a fresh complete growth medium containing the desired compound at final concentrations ranging from 1 μM to 100 μM with a ten-fold dilution step; 0.05% DMSO was used as a solvent control. After 48 h of drug treatment, the medium was discarded, the cells were washed with Dulbecco’s phosphate buffered saline with calcium and magnesium (DPBS) (Corning, Corning, NY, USA, Cat. No. 06821012), and 100 μL of 50 mM resazurin (Acros Organics, Geel, Belgium, Cat. No. A0428001) solution in DPBS was added to each well. Thereafter, the fluorescence was measured by a Synergy H1 Hybrid Multi-Mode Microplate Reader (BioTek Instruments, Inc., Winooski, VT, USA) using a 540 nm excitation and 590 nm emission filter. The three independent experiments were carried out in triplicates.

### 5.8. Real-Time Cytotoxicity Assay

The cytotoxic effect of the most active compounds was analyzed in real-time using the Agilent xCELLigence Real-Time Cell Analysis (RTCA) machine. Vero E6 cells were seeded on a 16-well E-plate (ACEA Biosciences, Inc., San Diego, CA, USA ) at a density of 5 × 10^3^ cells/well and allowed to adhere overnight. On the next day, the studied compounds were added at the final concentrations ranging from 0.078 μM to 5 μM with a two-fold dilution step for compounds **C1**, **C2**, and **C3**, and at the final concentrations ranging from 1.56 μM to 50 μM with a two-fold dilution step for compounds **C5** and **C6**; 0.01% DMSO was used as a solvent control. The cells were incubated for 5 days, and the signals between the electrodes (impedance) were recorded using an RTCA xCELLigence machine (Agilent Technologies, Inc., Santa Clara, CA, USA). The cell indexes corresponding to the 24 h of exposure were used to calculate the 50% cytotoxic concentration (CC_50_). The CC_50_ values were calculated using GraphPad Prism 10.0 for Windows, GraphPad Software, v. 10.4.1, Boston, MA, USA, www.graphpad.com (accessed on 16 February 2025).

### 5.9. Antiviral Activity

Vero E6 cells were seeded on 24-well tissue culture plates at a density of 5 × 10^4^ cells/well in 500 μL of DMEM and were allowed to adhere overnight. On the next day, cells were infected with the ZIKV-Nanoluc at 0.1 plaque-forming unit per cell (MOI 0.1) in a virus growth medium (VGM) (200 μL/well) containing DMEM, 0.2% bovine serum albumin (BSA), 100 IU penicillin/100 μg/mL streptomycin, and compounds at tested concentrations. After 1 h post-infection (hpi), a fresh growth medium (200 μL/well) containing DMEM, 2% heat-inactivated FBS, penicillin/streptomycin, and compounds at tested concentrations were added. The infected cells were treated with different concentrations of the compounds (or mock-treated with 0.05% DMSO). At 32 hpi for ZIKV-Nanoluc and 24 hpi for DENV2-Nanoluc, KUNV-Nanoluc, and TBEV-Nanoluc, the infectious medium was discarded, cells were lysed using passive lysis buffer (100 μL/well) (Promega, Madison, WI, USA), and the activity of nanoluciferase was measured using the Nano-Glo Luciferase Assay System (Promega).

The antiviral activity of compounds against DENV2-Nanoluc, KUNV-Nanoluc, and TBEV-Nanoluc assays were carried out according to the above-described protocol in a 96-well format with required modifications.

All assays were carried out three times in triplicates. EC_50_ calculation was performed using GraphPad Prism 10.0 for Windows, GraphPad Software, Boston, MA USA, www.graphpad.com.

### 5.10. Replicon Assays

Flaviviral replicons used in these assays encode for the viral 5′UTR, C protein, NanoLuc marker sequence, FMDV 2A sequence, codon-optimized C protein sequence, 30 codons of the E protein N-terminus, non-structural proteins’ coding sequence (NS1, NS2A, NS2B, NS3, NS4A, NS4B, NS5), and 3′ UTR (Figure S6). The sequences of all replicons used are given in fasta format in the Supporting information. Four flaviviral replicon’s encoding plasmids—ZIKV, KUNV, DENV2, and TBEV—with nanoluciferase reporter were linearized, and replicon RNAs were synthesized in vitro by using mMESSAGE mMACHINE SP6 Transcription Kit (Thermo Scientific, Cat. No. AM1340). Obtained replicon RNAs were purified by using the RNA Clean & Concentrator kit (Zymo Research, Irvine, CA, USA, Cat. No. R1017).

U2OS cells were seeded on 96-well tissue culture plates at a density of 1.5 × 10^4^ cells/well in 100 μL of IMDM and were allowed to adhere overnight. On the next day, the medium was replaced with a fresh complete growth medium, and 60 ng of replicon RNA were transfected per well following the commercial protocol of transfection reagent DharmaFECT II (Horizon Discovery, Waterbeach, UK, Cat. No. T-2002-03). After 1 h or 8 h post-transfection, the complete growth medium containing the desired compound was added directly onto the cells. After 32 h, the medium was discarded, cells were lysed, and the activity of nanoluciferase was measured using the Nano-Glo Luciferase Assay System (Promega). The three independent experiments were carried out in triplicates.

### 5.11. Protein Structures

The crystal structures of target proteins were downloaded from the Protein Data Bank (https://rcsb.org; accessed on 16 February 2025) [64]. The list of the protein structures used in the current work is given in Table 3. All raw crystal structures were prepared using the Schrödinger Protein Preparation Wizard [65,66] by adding missing hydrogen atoms, deleting the co-crystalized water, and capping protein termini with ACE and NMA residues.

### 5.12. Homology Modeling of TBEV NS3

Since the available crystal structures of the TBEV NS3 (PDB ID: 7OJ4) [74] lack protease domain containing catalytic triad (His-Asp-Ser), the homology modeling of the full-length TBEV NS3 was carried out using the SWISS-MODEL server [75]. The amino acid sequence of TBEV NS3 protease in FASTA format was retrieved from UniProt [76]. The SWISS-MODEL performs homology modeling using the following steps: (1) input data; (2) template search; (3) template selection; (4) model building; and (5) model quality estimation [75]. The generated 3D models of the NS3 protein with higher QMEAN and GMQE values [75] were selected for further minimization and modeling. The predicted 3D models were prepared using the Protein Preparation Wizard (OPLS_2005 force field [77]) in the Schrödinger LLC Maestro software [65]. Thereafter, the prepared structures were minimized using the MD simulation with a length of 50 ns using the Desmond simulation package of Schrödinger LLC [78], as described below. The quality of MD simulations was analyzed using the Simulation Quality Analysis tool implemented in the Desmond molecular dynamics package. The RMSD of the Cα atom positions in time was used to monitor the stability of the predicted structure of TBEV NS3 protein. The structures corresponding to the 50 ns time-point of the MD simulation were saved in a separate PDB file and used for further modeling.

### 5.13. Compound Structures Preparation

The two-dimensional structures (2D) of the studied compounds were downloaded in the structured data file (SDF) format from MolPort [58]. The ligand structures were prepared for molecular docking using LigPrep with the OPLS_2005 force field [77] from the Schrödinger Suite [79]. Generation of all possible states and ionization states was enumerated for each compound using Epik at a pH of 7.0 ± 2. The stereoisomers were determined from the three-dimensional (3D) structures. PDB files for the molecular docking procedure were created from the lowest-energy conformers for each compound.

### 5.14. Molecular Docking and Molecular Dynamics Simulations

AutoDock Vina 1.1.2 [43] was used for docking studies to find out the possible binding modes and binding energies of the studied compounds to the NS2B/NS3 proteases and NS5 RdRp of the studied viruses. For each NS3 protease, the binding site was specified with the catalytic triad residues (His51, Asp75, Ser135) and was surrounded by a grid box sized 25 × 25 × 25 points with a spacing of 1 Å. The docking parameters were used in their default values (one central processing unit to use, the number of output poses is 9, and the exhaustiveness is 8).

The molecular dynamics (MD) simulations were carried out using the Desmond program package of Schrödinger LLC [78,80]. The simulations were executed in a cubic simple point charge [81] water box using OPLS_2005 force field parameters [77]. Sodium and chloride ions were placed in the solvent to a concentration of 0.15 M; thereafter, to achieve electroneutrality, additional ions were added to the system. The isothermal–isobaric ensemble with a temperature of 300 K and a pressure of 1 bar was applied to all runs. The simulation length was 50 ns with a relaxation time of 1 ps for each run. The MD simulations results were analyzed using the simulation interaction diagram tool implemented in the Desmond molecular dynamics package.

### 5.15. Statistical Analysis

Statistical analysis was performed using Excel software (Microsoft Corp., Redmond, WA, USA). Data were analyzed using a one-way ANOVA test (parametric test). Results were considered statistically significant at p values lower than 0.05: * *p* < 0.05, ** *p* < 0.01, *** *p* < 0.001, **** *p* < 0.0001.

## Figures and Tables

**Table 2:** The AutoDock Vina 1.1.2 calculated binding energies, ligand efficiencies, and interactions of the compound **C3** with NS2B/NS3 or NS5 proteins of the studied viruses.

Virus	NS2B/NS3				NS5 RdRP			
	PDB ID	ΔG, kcal/mol	LE *	Interaction (*H-Bond*)	PDB ID	ΔG, kcal/mol	LE	Interaction (*H-Bond*)
DENV2	2FOM	−6.3	0.27	His 51, Leu128, Pro132, Ser135, Tyr150, *Gly151 (O(backbone)...HO)*, Tyr161	6IZX	−7.8	0.34	Leu462, *Arg729 (NH_2_…O (benzofuran)),* Arg737, *Gln742 (NH_2_…*^−^*OOC),* Ser757, Tyr758, Met761, *Thr790 (OH…*^−^*OOC)*, His801, Trp803
ZIKV	7OBV	−7.0	0.30	Ser135, Gly151, Asn152, *Gly153 (NH...O)*, Val154, Val155, Tyr161	6LD1	−7.3	0.31	*Arg459 (NH_2_…*^−^*OOC; NH_2_^+^…*^−^*OOC), Arg739 (NH_2_…O (benzofuran))*, Ser798, Ile799
KUNV	2IJO	−6.1	0.26	Ile18, Ile19, *Arg20 (NH(sidechain)…* ^−^*OOC)*, Thr27, Tyr34, Lys46, Asp51, Met52	2HCN	−5.9	0.26	Asn613, *Ser666 (OH…^−^OOC)*, Asp669, Cys714, Ser801
TBEV	-	−5.8	0.25	Leu118, Leu120, Thr122, Asn155, Leu157, *Ser166 (OH…OH)*, Ala168	7D6N	−6.5	0.28	Thr608, *Tyr609 (NH (backbone)…*^−^*OOC)*, Asn612, Ser662, Trp796, *Ser797 (OH…O (benzofuran carbonyl))*, *Ile798 (NH(backbone)…O(benzofuran carbonyl))*

* LE = ΔG/N_h_, where N_h_ is a number of heavy (non-hydrogen) atoms.

**Table 3:** The list of the protein structures used in the study.

PDB ID	Name of the Protein	Resolution, Å	Released	Ref.
6IZX	The RNA-dependent RNA polymerase domain of dengue 2 NS5, bound with RK-0404678	2.43	2019	[67]
6LD1	Zika NS5 polymerase domain	1.40	2020	[68]
2HCN	RNA-dependent RNA polymerase domain from West Nile Virus	2.35	2007	[69]
7D6N	Tick-borne encephalitis virus RNA-dependent RNA polymerase	3.17	2020	[70]
2FOM	Dengue virus 2 NS2B/NS3 protease	1.5	2006	[71]
7OBV	Zika virus protease in complex with inhibitor MI-2248	1.30	2022	[72]
2IJO	West Nile virus NS2B-NS3 protease complexed with bovine pancreatic trypsin inhibitor	2.30	2007	[73]

## Data Availability

The data that support the findings of this study are available on request from the corresponding authors, E.Z. (eva.zusinaite@ut.ee) and M.K. (mati.karelson@ut.ee). Samples of the compounds are available from the authors.

## Supplementary Materials

The following supporting information can be downloaded at: https://www.mdpi.com/article/10.3390/ph18030283/s1, Table S1. The summarized data on the ChEMBL dataset; Table S2. Primers used in CPER reactions with different flaviviruses; Figure S1. Multiple sequence alignment of NS5 protein from studied flaviviruses (DENV2 (Q8B489), KUNV (AAP78941), TBEV (AAB53095), ZIKV (AMA12085)); Figure S2. The effect of compounds **C1** and **C2** on Vero E6 cells assessed using xCELLigence Real-Time Cell Analysis; Figure S3. The effect of compounds **C3** and **C5** on Vero E6 cells assessed using xCELLigence Real-Time Cell Analysis; Figure S4. Calculated binding modes of compound **C3** at potential binding site of (A) DENV2 NS3 (PDB ID: 2FOM); (B) ZIKV NS3 (PDB ID: 7OBV); (C) WNV NS3 (PDB ID: 2IJO); (D) TBEV NS3 (full-length structure generated by SWISS-MODEL (https://swissmodel.expasy.org/)); Figure S5. Calculated binding modes of compound **C3** at potential binding site of (A) DENV2 NS5 (PDB ID: 6IZX); (B) ZIKV NS5 (PDB ID: 6LD1); (C) WNV NS5 (PDB ID: 2HCN); (D) TBEV NS5 (PDB ID: 7D6N). Figure S6. RMSD of the atomic positions for the compound **C3** and NS5 RdRp of studied viruses of the 50 ns MD simulations using the Desmond package of Schrödinger: (A) DENV2 NS5 (PDB ID: 6IZX); (B) ZIKV NS5 (PDB ID: 6LD1); (C) WNV NS5 (PDB ID: 2HCN); (D) TBEV NS5 (PDB ID: 7D6N); Figure S7. RMSD of the atomic positions for the compound **C3** and NS3 of studied viruses of the 50 ns MD simulations using the Desmond package of Schrödinger: (A) DENV2 NS3 (PDB ID: 2FOM); (B) ZIKV NS3 (PDB ID: 7OBV); (C) WNV NS3 (PDB ID: 2IJO); (D) TBEV NS3 (full-length structure generated by SWISS-MODEL (https://swissmodel.expasy.org/)); Figure S8. The schematic representation of flavivirus replicons constructed in this study. The sequences of all studied flavivirus replicons in fasta format. Animated MD simulation trajectory for DENV2 NS5–**C3** complex.
