# Supplementary material for: Dengue Virus Inhibitors as Potential Broad-Spectrum Flavivirus Inhibitors
Source: Pharmaceuticals (Basel). 2025 Feb 20;18(3):283. doi: 10.3390/ph18030283 (PMC11944514; doi:10.3390/ph18030283)
Supplement: Supplementary file 1 [file pharmaceuticals-18-00283-s001.zip › Supporting_Information_R3.docx]

**Dengue virus inhibitors as potential broad-spectrum flavivirus inhibitors**

# Larisa Ivanova^1,+^, Krystyna Naumenko^2,3+^, Margus Varjak^4,+^, Sandra Koit^2^, Yehudit Morozovsky^1^, Andres Merits^2^, Mati Karelson^1^, Eva Zusinaite^2,*^

**Supporting Information**

**Table S1.** The summarized data on the ChEMBL data set

| **Target ChEMBL ID** | **Target name** | **Compound ChEMBL ID** | **EC_50_, nM** | **MolPort code** | **Similarity index*** | **Code** |
| --- | --- | --- | --- | --- | --- | --- |
| CHEMBL5980 | DENV-2 NS3 protein | CHEMBL1353221 | 1610 | MolPort-000-716-514 | 100 | **C8** |
|  |  | CHEMBL1570587 | 3530 | MolPort-002-549-069 | 100 | - |
|  |  | CHEMBL1707222 | 3770 | MolPort-028-915-505 | 80 | - |
|  |  | CHEMBL1706454 | 4580 | MolPort-000-794-330 | 83 | - |
|  |  | CHEMBL1972037 | 4590 | MolPort-005-892-540 | 100 | - |
|  |  | CHEMBL2006418 | 4600 | MolPort-004-248-471 | 100 | - |
|  |  | CHEMBL1699845 | 4630 | MolPort-001-846-896 | 100 | - |
|  |  | CHEMBL1496231 | 4670 | MolPort-002-559-633 | 100 | - |
|  |  | CHEMBL131037 | 5230 | MolPort-003-662-179 | 90 | - |
|  |  | CHEMBL1329974 | 5400 | MolPort-000-644-465 | 100 | - |
|  |  | CHEMBL1702945 | 5470 | MolPort-002-323-291 | 84 | - |
|  |  | CHEMBL1325819 | 5640 | MolPort-000-233-992 | 100 | - |
|  |  | CHEMBL1302524 | 5890 | MolPort-002-151-527 | 91 | - |
|  |  | CHEMBL1721095 | 5910 | MolPort-002-134-455 | 100 | - |
|  |  | CHEMBL1347373 | 6040 | MolPort-002-241-109 | 100 | - |
|  |  | CHEMBL3191525 | 6250 | MolPort-001-971-001 | 96 | - |
|  |  | CHEMBL1526763 | 6330 | MolPort-002-221-181 | 100 | - |
|  |  | CHEMBL1163377 | 6330 | MolPotr-000-794-330 | 100 | - |
|  |  | CHEMBL1526067 | 6370 | MolPort-002-706-222 | 89 | - |
|  |  | CHEMBL1736432 | 6380 | MolPort-019-777-354 | 89 | - |
|  |  | CHEMBL1721986 | 6890 | MolPort-000-645-509 | 100 | - |
|  |  | CHEMBL1476089 | 7020 | MolPort-035-758-559 | 100 | - |
|  |  | CHEMBL1564699 | 7150 | MolPort-000-627-415 | 100 | - |
|  |  | CHEMBL1383957 | 7170 | MolPort-001-524-700 | 100 | - |
|  |  | CHEMBL1465938 | 7220 | MolPort-000-627-622 | 100 | **-** |
|  |  | CHEMBL1433919 | 7410 | MolPort-018-528-550 | 100 | **-** |
|  |  | CHEMBL1420642 | 7440 | MolPort-001-662-328 | 100 | **-** |
|  |  | CHEMBL1310865 | 7490 | MolPort-000-682-974 | 100 | **-** |
|  |  | CHEMBL1321103 | 7650 | MolPort-004-247-780 | 100 | **-** |
|  |  | CHEMBL1331211 | 7660 | MolPort-000-682-969 | 100 | **-** |
|  |  | CHEMBL1578997 | 7760 | MolPort-000-792-813 | 100 | **-** |
|  |  | CHEMBL1873953 | 7790 | MolPort-019-909-929 | 86 | **-** |
|  |  | CHEMBL1528850 | 7970 | MolPort-002-238-858 | 100 | **-** |
|  |  | CHEMBL1881398 | 7990 | MolPort-007-557-304 | 100 | **-** |
|  |  | CHEMBL1431126 | 8260 | MolPort-019-692-401 | 99 | **-** |
|  |  | CHEMBL1507776 | 8420 | MolPort-019-728-143 | 100 | **-** |
|  |  | CHEMBL1531656 | 8550 | MolPort-000-808-935 | 100 | **-** |
|  |  | CHEMBL1723955 | 9150 | MolPort-002-559-421 | 100 | **-** |
|  |  | CHEMBL1865393 | 9200 | MolPort-007-565-717 | 90 | **-** |
|  |  | CHEMBL1486234 | 9490 | MolPort-001-013-886 | 94 | **-** |
|  |  | CHEMBL1538386 | 9500 | MolPort-002-705-122 | 100 | **-** |
|  |  | CHEMBL1607263 | 9530 | MolPort-000-682-973 | 100 | **-** |
|  |  | CHEMBL3196407 | 9600 | MolPort-002-113-998 | 100 | **-** |
|  |  | CHEMBL1319867 | 9700 | MolPort-000-711-534 | 100 | **C5** |
|  |  | CHEMBL1360792 | 9850 | MolPort-002-706-222 | 100 | **-** |
| CHEMBL613966 | DENV2 | CHEMBL2042322 | 7.4 | MolPort-039-333-202 | 89 | **C2** |
|  |  | CHEMBL3426587 | 6 | MolPort-008-351-867 | 100 | **C6** |
|  |  | CHEMBL3426586 | 11 | MolPort-008-351-867 | 100 | **-** |
|  |  | CHEMBL3426543 | 12 | MolPort-008-353-064 | 100 | **-** |
|  |  | CHEMBL3426548 | 21 | MolPort-008-352-646 | 97 | **-** |
|  |  | CHEMBL3426547 | 25 | MolPort-008-352-646 | 97 | **-** |
|  |  | CHEMBL343286 | 29 | MolPort-002-910-067 | 91 | **C4** |
|  |  | CHEMBL341590 | 36 | MolPort-003-804-636 | 93 | **-** |
|  |  | CHEMBL3422595 | 39 | MolPort-002-910-070 | 91 | - |
|  |  | CHEMBL3426576 | 42 | MolPort-008-353-064 | 93 | - |
|  |  | CHEMBL3426589 | 50 | MolPort-008-352-754 | 96 | - |
|  |  | CHEMBL3426592 | 51 | MolPort-008-353-064 | 100 | - |
|  |  | CHEMBL402274 | 80 | MolPort-003-804-636 | 92 | - |
|  |  | CHEMBL1630220 | 100 | MolPort-035-765-986 | 79 | - |
|  |  | CHEMBL866 | 700 | MolPort-006-111-411 | 100 | **C3** |
|  |  | CHEMBL116438 | 4200 | MolPort-003-665-670 | 100 | **-** |
|  |  | CHEMBL1197690 | 5000 | MolPort-002-579-851 | 100 | **-** |
|  |  | CHEMBL1269736 | 5460 | MolPort-001-785-903 | 87 | **C7** |
| CHEMBL613757 | DENV | CHEMBL1643 | 49 | MolPort-003-724-722 | 100 | **-** |
|  |  | CHEMBL1652120 | 75 | MolPort-039-015-418 | 79 | **-** |
|  |  | CHEMBL1652118 | 100 | MolPort-023-276-901 | 78 | **-** |
|  |  | CHEMBL311226 | 1000 | MolPort-003-845-673 | 100 | **-** |
|  |  | CHEMBL82242 | 1700 | MolPort-000882-121 | 100 | **-** |
|  |  | CHEMBL471282 | 1700 | MolPort-021-804-572 | 100 | **-** |
|  |  | CHEMBL402947 | 1700 | MolPort-006-069-272 | 100 | **-** |
|  |  | CHEMBL402947 | 1700 | MolPort-006-069-272 | 100 | **-** |
|  |  | CHEMBL251254 | 1700 | MolPort-001-740-566 | 100 | **-** |
|  |  | CHEMBL269277 | 1700 | MolPort-003-939-279 | 100 | **-** |
|  |  | CHEMBL3604361 | 7100 | MolPort-035-395-169 | 91 | **-** |
| CHEMBL613728 | DENV4 | CHEMBL98745 | 49 | MolPort-019-939-284 | 100 | **-** |
|  |  | CHEMBL487498 | 203 | MolPort-019-939-284 | 96 | **-** |
|  |  | CHEMBL400092 | 835 | MolPort-000-882-129 | 100 | **C1** |
|  |  | CHEMBL586091 | 1348 | Molport-044-754-200 | 100 | **-** |
|  |  | CHEMBL251056 | 1705 | MolPort-002-526-469 | 80 | **-** |
|  |  | CHEMBL98481 | 2166 | MolPort-002-526-469 | 82 | **-** |

***** A similarity index lower than 100 means that the original CHEMBL compound was not commercially available and was replaced by an analogue with an indicated similarity index.

**Table S2.** Primers used in CPER reactions with different flaviviruses.

| **CPER primers for DENV2** | |
| --- | --- |
| Primer pair I | GCTCGTTTAGTGAACCGAGTTGTTAGTCTACGTGGACC |
|  | GTTGTCGACCTGCCCATGTCC |
| Primer pair II | GGACATGGGCAGGTCGACAAC |
|  | GTCTCTCCTATGTTGCCAGTTCC |
| Primer pair III | GGAACTGGCAACATAGGAGAGAC |
|  | ATGCCATGCCGACCCGAACCTGTTGATTCAACAGCAC |
| Primer pair IV | GTGCTGTTGAATCAACAGGTTCT GGGTCGGCATGGCAT |
|  | GGTCCACGTAGACTAACAACT CGGTTCACTAAACGAGC |
| **CPER primers for KUNV** | |
| Primer pair I | GCTCGTTTAGTGAACCGAGTAGTTCGCCTGTGTG |
|  | CAATCATGTCAGCGTTGTAGGCATTCACCTGTGACTGCA |
| Primer pair II | TGCAGTCACAGGTGAATGCCTACAACGCTGACATGATTG |
|  | GTCCTTTTGCCCCACCTCTCTTCAGCCCTGGTTTTTC |
| Primer pair III | GAAAAACCAGGGCTGAAGAGAGGTGGGGCAAAAGGAC |
|  | ATGCCATGCCGACCCAGATCCTGTGTTCTCGCAC |
| Primer pair IV | GTGCGAGAACACAGGATCTGGGTCGGCATGGCAT |
|  | CACACAGGCGAACTACTCGGTTCACTAAACGAGC |
| **CPER primers for TBEV** | |
| Primer pair I | GCTCGTTTAGTGAACCGAGATTTTCTTGCACGTGCATG |
|  | CCTCACTAAGTAATTCACCGTTGTC |
| Primer pair II | GACAACGGTGAATTACTTAGTGAGG |
|  | GTGTCTCCCTCAGAACCACC |
| Primer pair III | GGTGGTTCTGAGGGAGACAC |
|  | ATGCCATGCCGACCCAGCGGGTGTTTTTCCGAGTC |
| Primer pair IV | GACTCGGAAAAACACCCGCTGGGTCGGCATGGCAT |
|  | CATGCACGTGCAAGAAAATCTCGGTTCACTAAACGAGC |


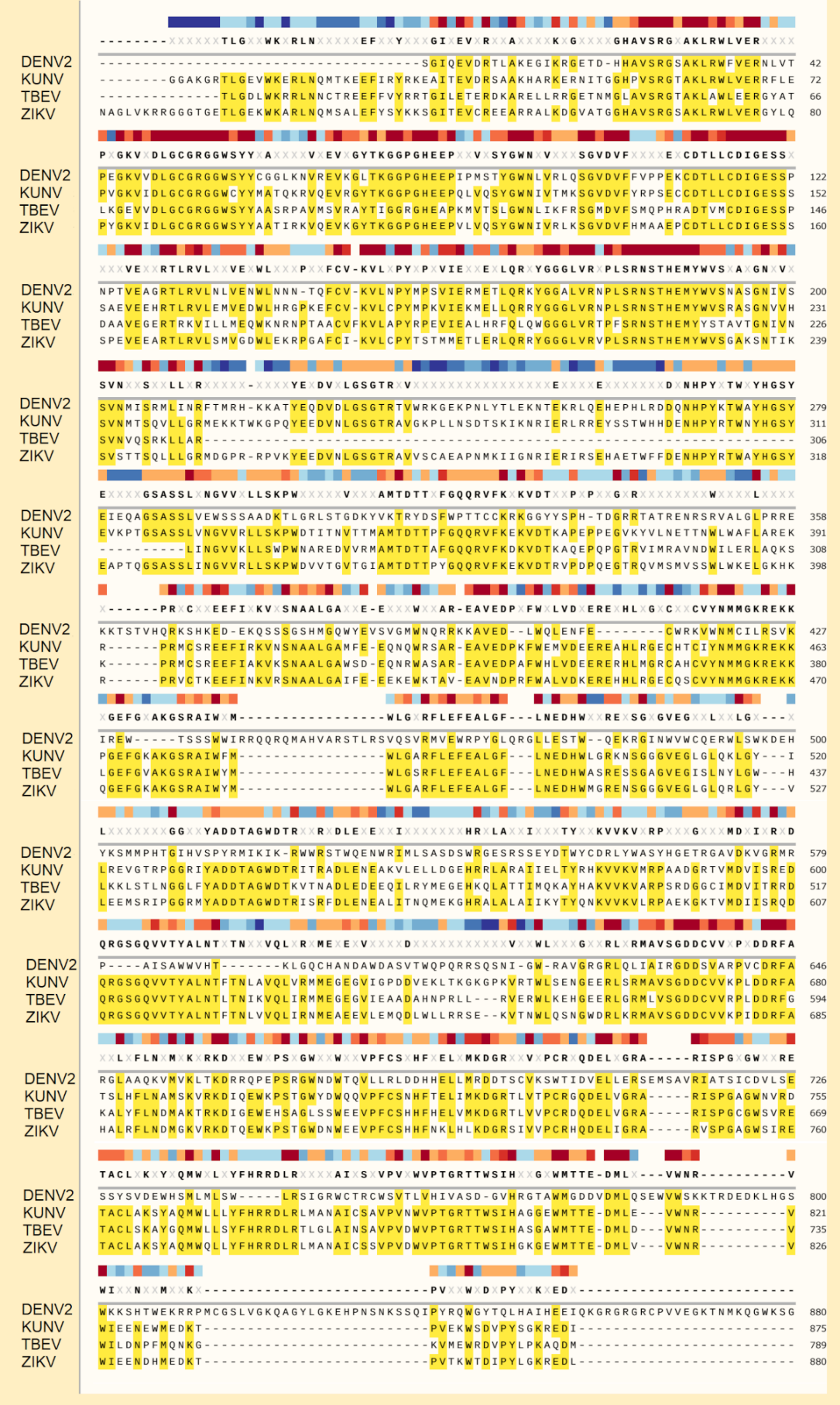


**Figure S1.** Multiple sequence alignment of NS5 protein from studied flaviviruses (DENV2 (Q8B489), KUNV (AAP78941), TBEV (AAB53095), ZIKV (AMA12085)). The alignment was done using MUSCLE software.


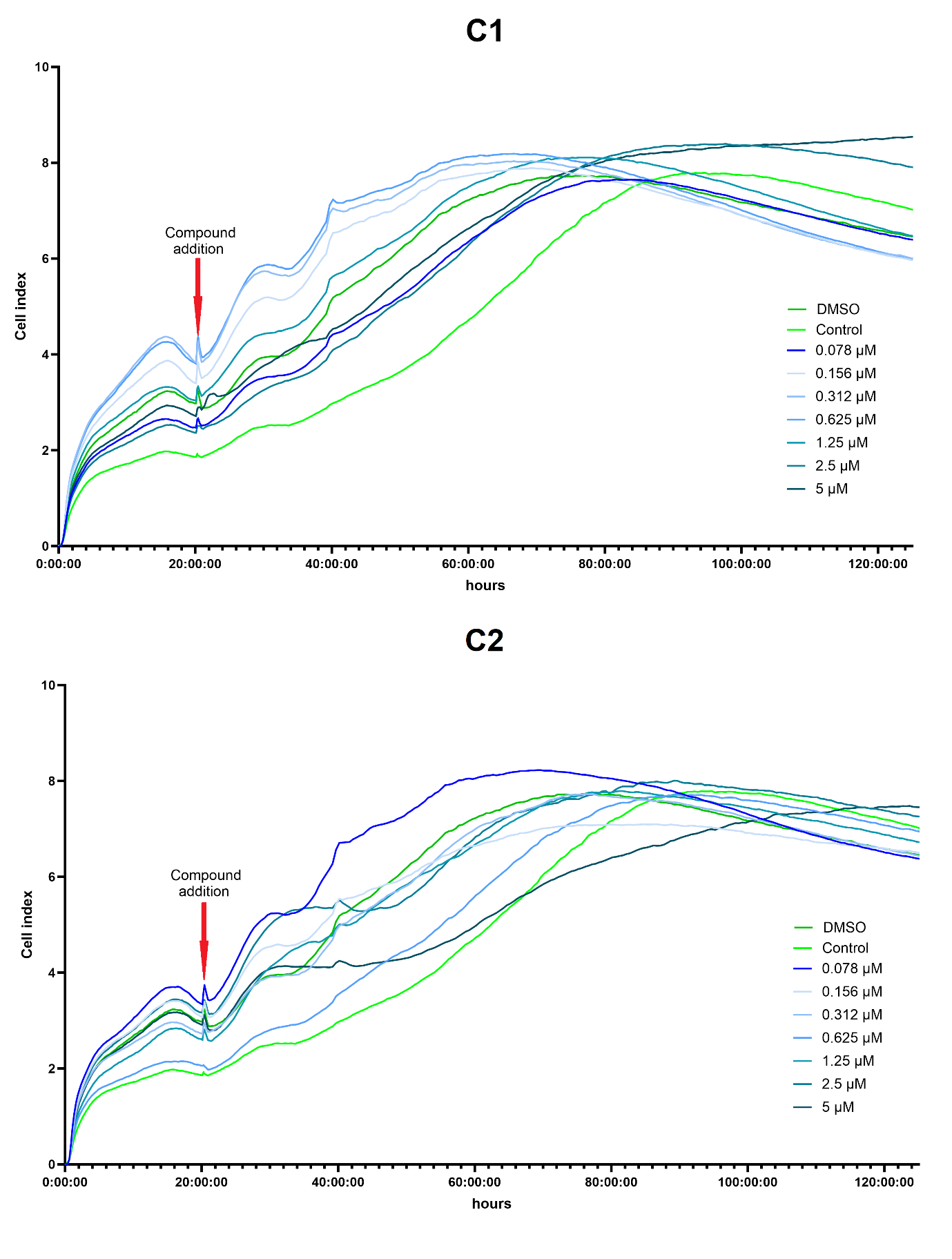


**Figure S2.** The effect of compounds **C1** and **C2** on Vero E6 cells assessed using xCELLigence Real-Time Cell Analysis (RTCA).


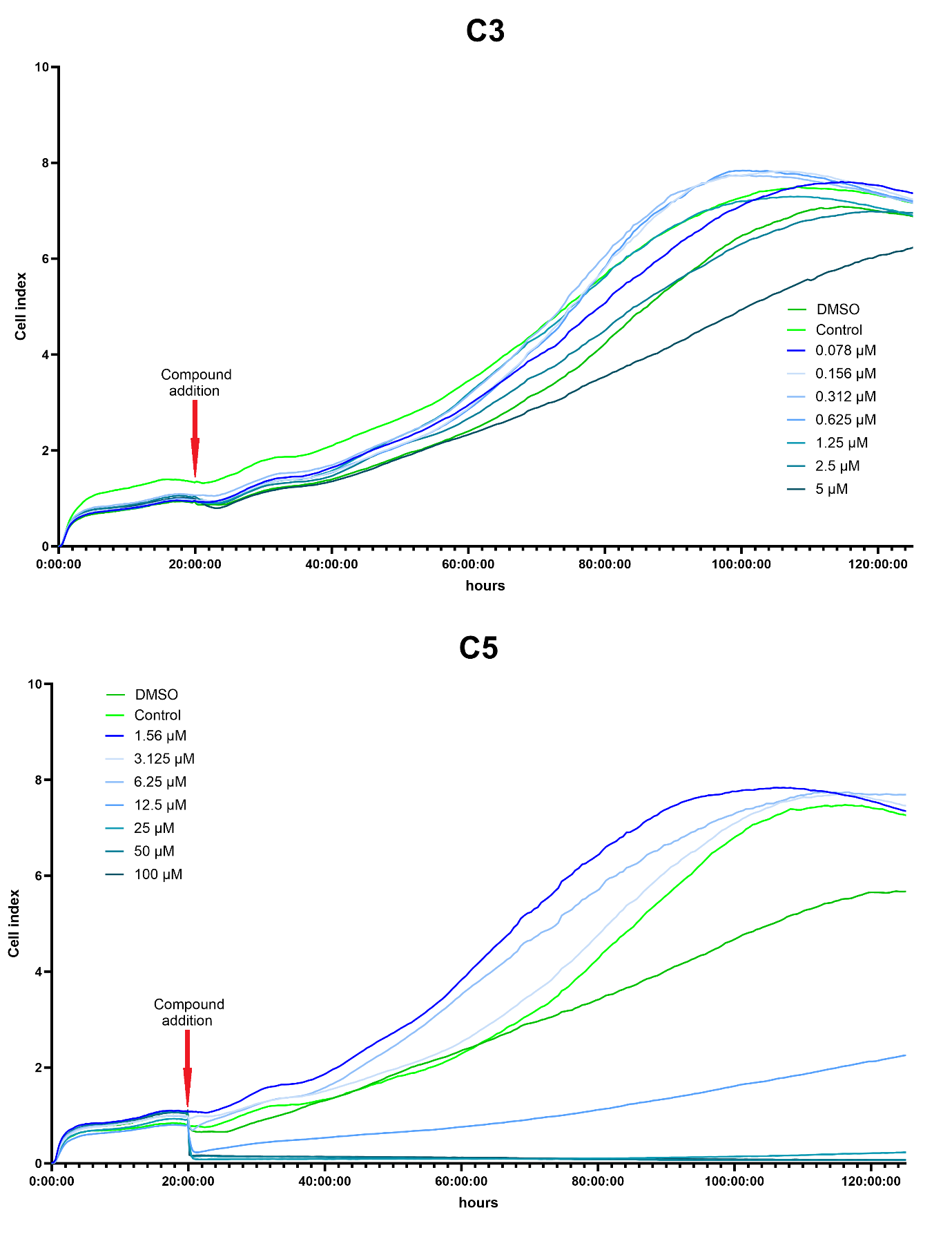


**Figure S3.** The effect of compounds **C3** and **C5** on Vero E6 cells assessed using xCELLigence Real-Time Cell Analysis (RTCA).


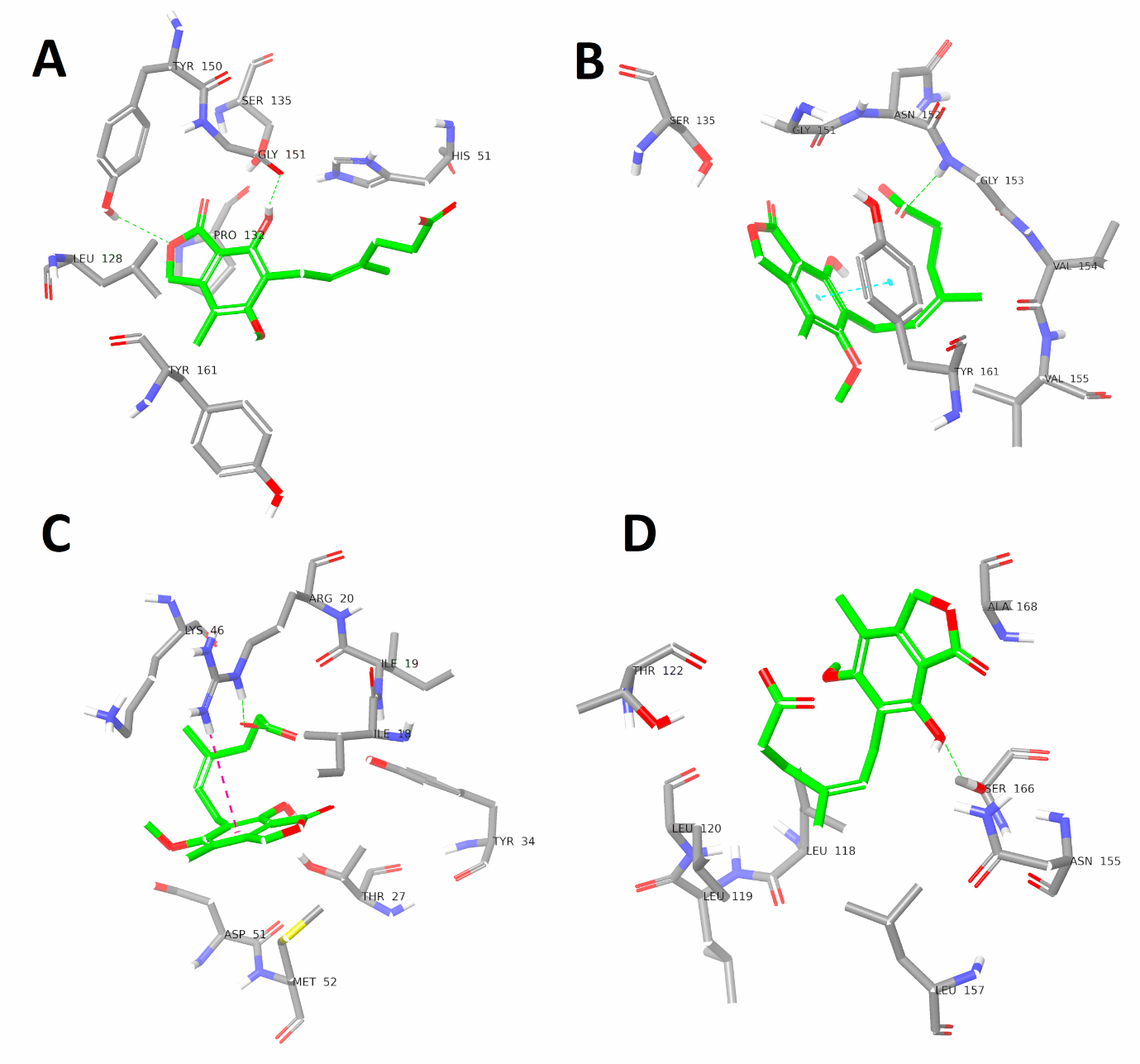


**Figure S4.** Calculated binding modes of compound **C3** at potential binding site of (**A**) DENV2 NS3 (PDB ID: 2FOM); (**B**) ZIKV NS3 (PDB ID: 7OBV); (**C**) WNV NS3 (PDB ID: 2IJO); (**D**) TBEV NS3 (full-length structure generated by SWISS-MODEL (https://swissmodel.expasy.org/)). Intermolecular hydrogen bonds are shown by green dashed lines. The pi-pi and pi-cation interactions are shown by blue and pink dashed lines, respectively.


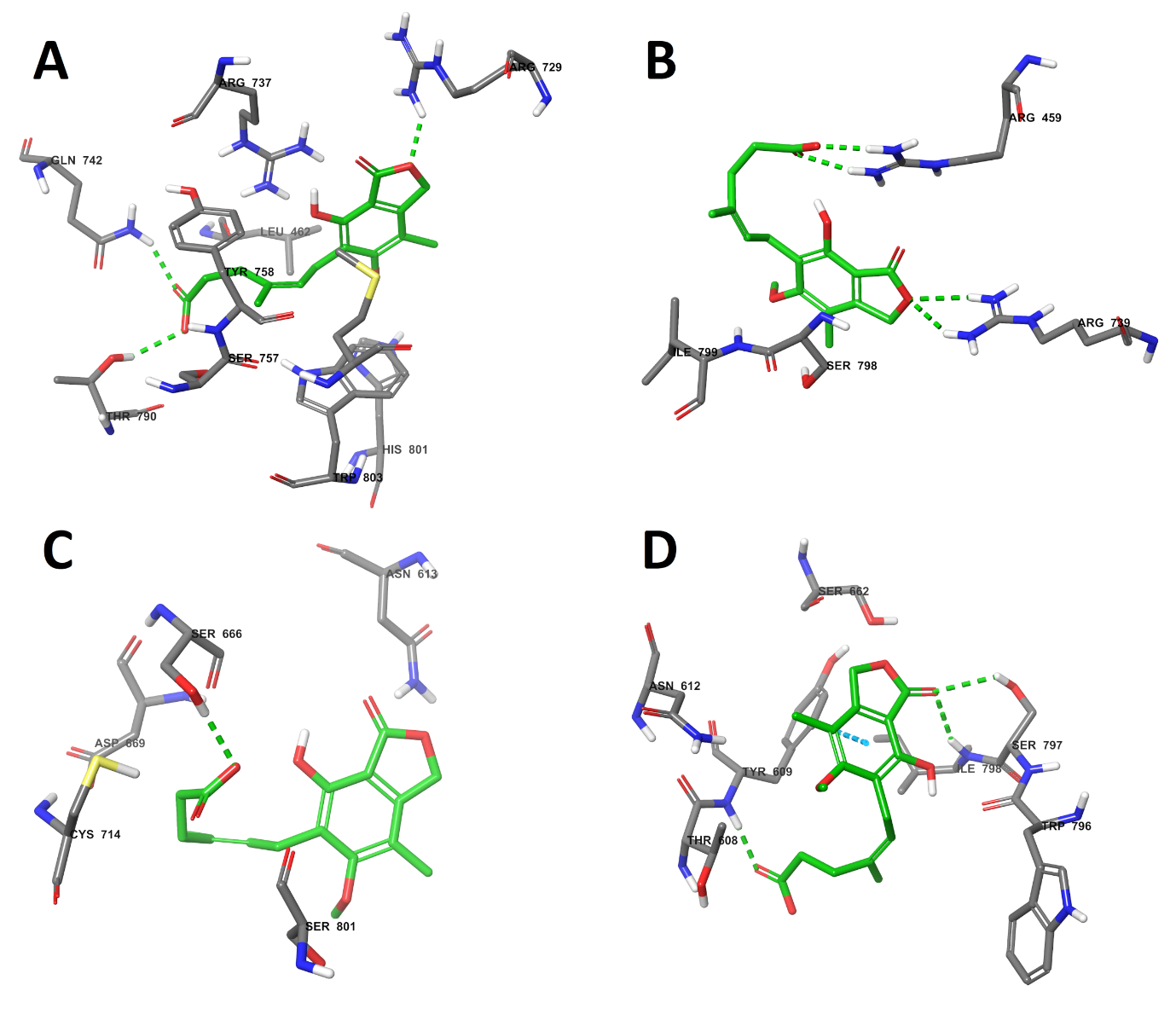


**Figure S5.** Calculated binding modes of compound **C3** at potential binding site of (**A**) DENV2 NS5 (PDB ID: 6IZX); (**B**) ZIKV NS5 (PDB ID: 6LD1); (**C**) WNV NS5 (PDB ID: 2HCN); (**D**) TBEV NS5 (PDB ID: 7D6N). Intermolecular hydrogen bonds are shown by green dashed lines. The pi-pi interactions are shown by blue dashed lines.


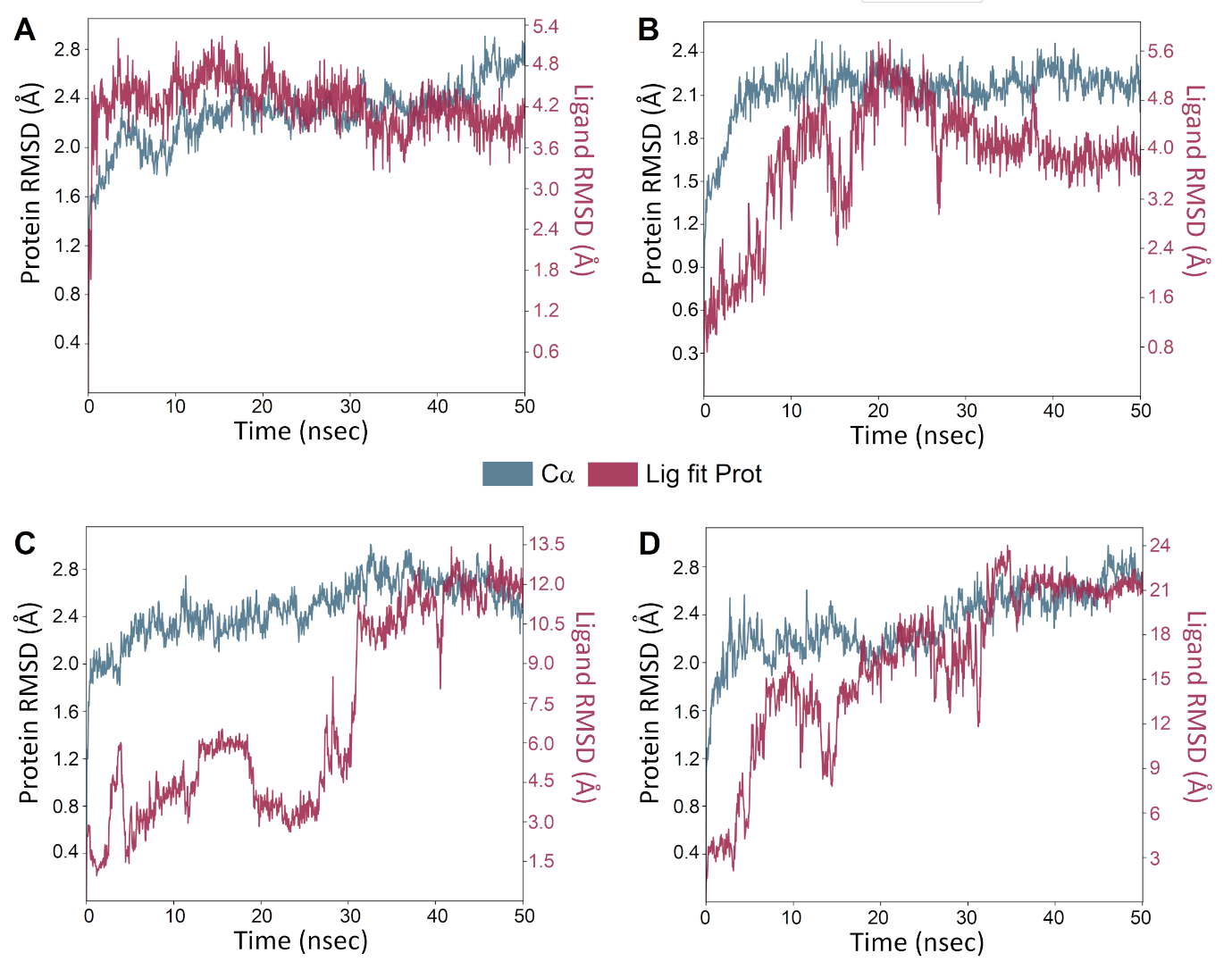


**Figure S6.** RMSD of the atomic positions for the compound **C3** (in red, Lig fit Prot) and NS5 RdRp of studied viruses (Cα positions in blue) of the 50 ns MD simulations using the Desmond package of Schrödinger: (**A**) DENV2 NS5 (PDB ID: 6IZX); (**B**) ZIKV NS5 (PDB ID: 6LD1); (**C**) WNV NS5 (PDB ID: 2HCN); (**D**) TBEV NS5 (PDB ID: 7D6N).


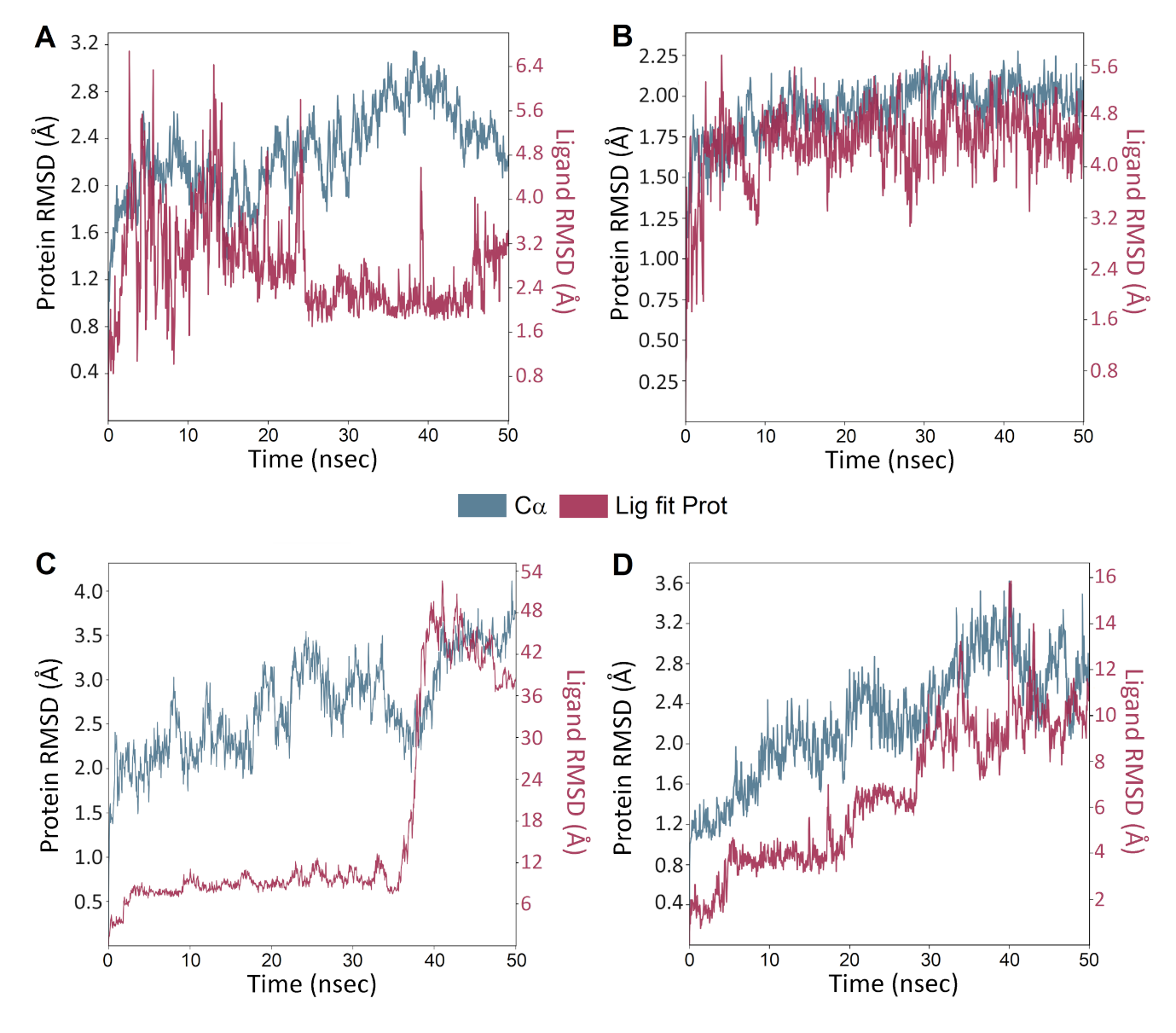


**Figure S7.** RMSD of the atomic positions for the compound **C3** (in red, Lig fit Prot) and NS3 of studied viruses (Cα positions in blue) of the 50 ns MD simulations using the Desmond package of Schrödinger: (**A**) DENV2 NS3 (PDB ID: 2FOM); (**B**) ZIKV NS3 (PDB ID: 7OBV); (**C**) WNV NS3 (PDB ID: 2IJO); (**D**) TBEV NS3 (full-length structure generated by SWISS-MODEL (https://swissmodel.expasy.org/)).


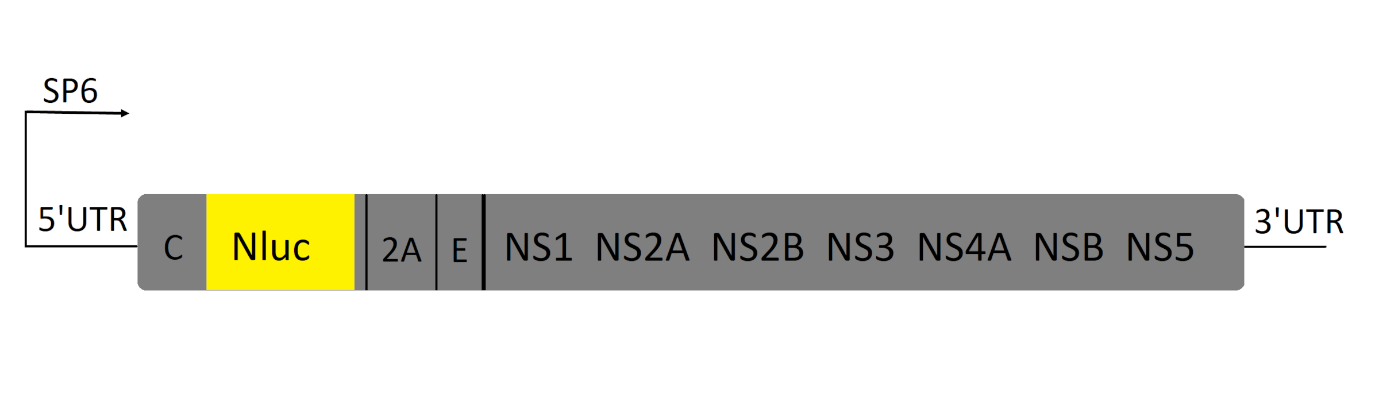


**Figure S8.** The schematic representation of flavivirus replicons constructed in this study.
